# An Integrated Self-Powered Photoelectrocatalytic System for Quadruple-Function Water Remediation: Organic Degradation, Radionuclide Recycling, Energy Recovery, and Toxicity Evaluation

**DOI:** 10.3390/ijms27198680

**Published:** 2026-09-28

**Authors:** Sivakumar Vigneshwaran, Shi-Cheng Wang, Qinhe Pan

**Affiliations:** 1 Key Laboratory of Advanced Materials of Tropical Island Resources, Ministry of Education, School of Chemistry and Chemical Engineering, Hainan University, Haikou 570228, China; drsvignesh@hainanu.edu.cn; 2 State Key Laboratory of Inorganic Synthesis and Preparative Chemistry, College of Chemistry, Jilin University, Changchun 130012, China; 3 National Key Laboratory of Uranium Resources Exploration-Mining and Nuclear Remote Sensing, East China University of Technology, Nanchang 330013, China

**Keywords:** complex radioactive wastewater, modified carbon felt, uranium recovery, organic degradation, electricity production, self-driven PEC system, phytotoxicity

## Abstract

The pervasive prevalence of organic pollutants in water bodies presents major obstacles to traditional uranic recovery methods. Herein, a novel self-powered catalytic system has been developed, featuring a TGCNM heterojunction cathode paired with a carbon/chitin nanofilm nanoarray (CNF) and a silicon PVC photovoltaic (Si PVC) photoanode. This advanced assembly enables the concurrent reduction of U(VI), degradation of pollutants, and generation of electricity. The synergy between the functional groups and redox-active sites on the TGCNM heterojunction promotes efficient adsorption and stepwise reduction of U(VI), granting the system remarkable cycling stability and promising real-world applicability. When exposed to simulated sunlight, the system demonstrates impressive removal rates: 96.7% for UO_2_^2+^ and 97.7% for bisphenol A (BPA). Additionally, U(VI) removal remains above 90.2% in various organic environments, illustrating strong resistance to interference. Even after five consecutive cycles, the system retains high performance (>93.9% for U(VI) and >94.3% for BPA). Under actual solar irradiation, removal efficiencies exceed 95.8% for both contaminants, underscoring its practical value. This study offers innovative perspectives on resource-focused strategies for remediating radioactive and organic-contaminated waters by integrating energy production and water treatment.

## 1. Introduction

The transport and transformation mechanisms of uranium in aquatic environments have garnered significant attention due to its strategic value, and the fact that it poses radiation threats in addition to the toxicity of heavy metals [1]. Uranium pollutants in the water system in the uranyl complexed state are prevalent due to processes including radioactive wastewater leaks, spent fuel reprocessing, and industrial uranium smelting [2]. The endocrine-disrupting activity, genotoxicity, and carcinogenicity of bisphenol A (BPA), 2,2-bis (4-hydroxyphenyl) propane, a commonly used industrial raw material, have also attracted substantial attention. Contaminated wastewater and natural water sources often contain BPA. Alternatively, uranium(VI) easily forms water-soluble UO_2_^2+^-L species with amino-polycarboxylates/humics when it coexists with organic ligands (e.g., surfactants, organophosphates) in a composite co-contamination regime [3]. The formation of stable uranium–organic complexes is one way in which this coordination effect increases the chemical stability of uranium species. However, there are two unintended consequences of this effect: Traditional adsorbents have their adsorption active sites sterically hindered by organic ligands [4], and the removal efficiency of monoprocesses (such as ion exchange or surface complexation) is impaired by coordination-stabilised uranium complexes [5]. As a result, treating deep nuclear wastewater and recycling uranium both require technologies that work together to degrade organic pollutants and recover uranium.

Due to its environmentally friendly and long-lasting properties, photocatalytic technology has recently gained attention as a promising new approach to cleaning up water systems that have been polluted with heavy metals and organic contaminants [6]. The core mechanism involves photoinduced charge separation in semiconductors (e.g., zinc oxide (ZnO), graphitic carbon nitride (g-C_3_N_4_)): photons with energy > bandgap excite valence electrons to the conduction band, yielding reductive electrons (e^−^) and oxidative holes (h^+^) [7]. Using e^−^, these two components work together to convert U(VI) to low toxicity U(IV) hydroxides, and h^+^ generated reactive oxygen species (ROS) (•OH/•O_2_^−^) destroy organics [8]. Some important restrictions include: (1) the use of UV-limited catalysts (<40%) and the fact that traditional methods, such as external bias and hole scavengers, come with costs and contamination dangers. New electrocatalytic approaches enhance charge separation, but they aren’t scalable since they need external power [9]. Furthermore, the effectiveness of photocatalytic reduction is decreased due to uranium–organic chelates (such as –OH/–COOH groups) interfering with electron transport and U valence conversion being hindered by their spatial blocking [10]. To overcome this obstacle in composite pollution cleanup, new self-driven catalytic structures incorporating ligand modulation and electron transport optimisation must be developed.

Recent research has focused on solar-powered photocatalytic fuel cells (PFCs) as a means of dual environmental cleanup under light, with the added benefit of recovering heavy metal ions and decomposing organic contaminants by photoelectrocatalytic redox [11]. A photoanode–cathode system that is powered by sunlight makes up the system. The photo anode creates electron-hole pairs when light separates their charges. These electrons are then transmitted to the cathode through an external circuit, where they are subjected to a Fermi potential gradient, allowing them to generate electricity [12]. The results suggest a plausible electron transfer pathway in which the cathode has multiple uses; one of them is producing energy, and another is dissolving heavy metal ions in wastewater [13]. For example, Liu et al. designed a TiO_2_ nanotube/graphite PFC that reduced Cr(VI) → Cr(III), mineralised methylene blue, and delivered consistent power [14]. Similarly, Lam et al. engineered a Z-scheme MoO_3_/ZnO heterojunction PFC enabling simultaneous phenol mineralisation, Cu^2+^ → Cu reduction, and stable power generation via directional charge separation [15]. Although optimising the anode is the main focus of current PFC research paradigms, little is known about how cathode alteration controls the efficiency of U(VI) removal through interfacial electron transport pathways. In order to create a bifunctional PFC platform for organic pollutant degradation and synergistic uranium immobilisation, this study pioneers the design of a hetero-junction-engineered cathode.

Efficient photoelectrocatalytic uranium extraction is achieved through the incorporation of hierarchical porosity and controllable electronic states into carbons generated from metal organic frameworks (MOFs) through controlled pyrolysis [16]. Together, heteroatom doping (N/S) and defect engineering improve uranium adsorption, and interconnected pores improve adsorption reduction kinetics by optimising charge transfer [17]. Through improved electron transport and adsorption, GCN increases U(VI) reduction by utilising metallic conductivity and nitrogen-rich surfaces. Uranium extraction is hindered by its low catalytic effectiveness due to interfacial charge recombination and weak carrier mobility [18]. Given MXene’s high surface area, variable surface chemistry, and metallic conductivity, rational composite design becomes possible, allowing for the resolution of this issue. By subjecting the GCN-MXene system to heat, two improvements are brought about: (1) the formation of conductive routes through in-situ carbon graphitisation, and (2) the exposure of high-index catalytic facets in defect-repaired GCN. The dielectric confinement of the carbon framework enhances light utilisation, and the 3D interfacial network that results from guided electron transfer inhibits charge recombination [19]. In this study, we created an ALCE, or autonomous light-driven catalytic ensemble, to convert energy and extract uranium at the same time. The system features a Ti_3_C_2_@GCN heterojunction cathode fabricated by coupling Ti_3_C_2_ MXene with GCN precursors followed by controlled thermal treatment, which provides abundant catalytic interfaces for efficient U(VI) reduction and adsorption. A photoelectroanode combines a back Si PVC solar cell with CNF that has been developed on carbon/chitin nano film seeded fluorine doped tin oxide (FTO) glass. CNF boosts the internal potential by absorbing photons with shorter wavelengths and transmitting them with longer ones. The study aims to demonstrate the following: first, through controlled experiments, the performance of ALCE in uranium reduction, organic degradation, and power generation; second, mechanistic elucidation of the reduction/degradation pathways; third, parametric investigation of influencing factors including electrolyte concentration, pH value, initial concentrations of organic/U(VI) contaminants, and co-existing ionic species; and finally, through cycling stability tests, simulated seawater experiments, multi-organic system evaluations, and real sunlight illumination trials, a comprehensive assessment of practical applicability.

## 2. Results and Discussion

### 2.1. Material Characterisation

**Figure 1:**
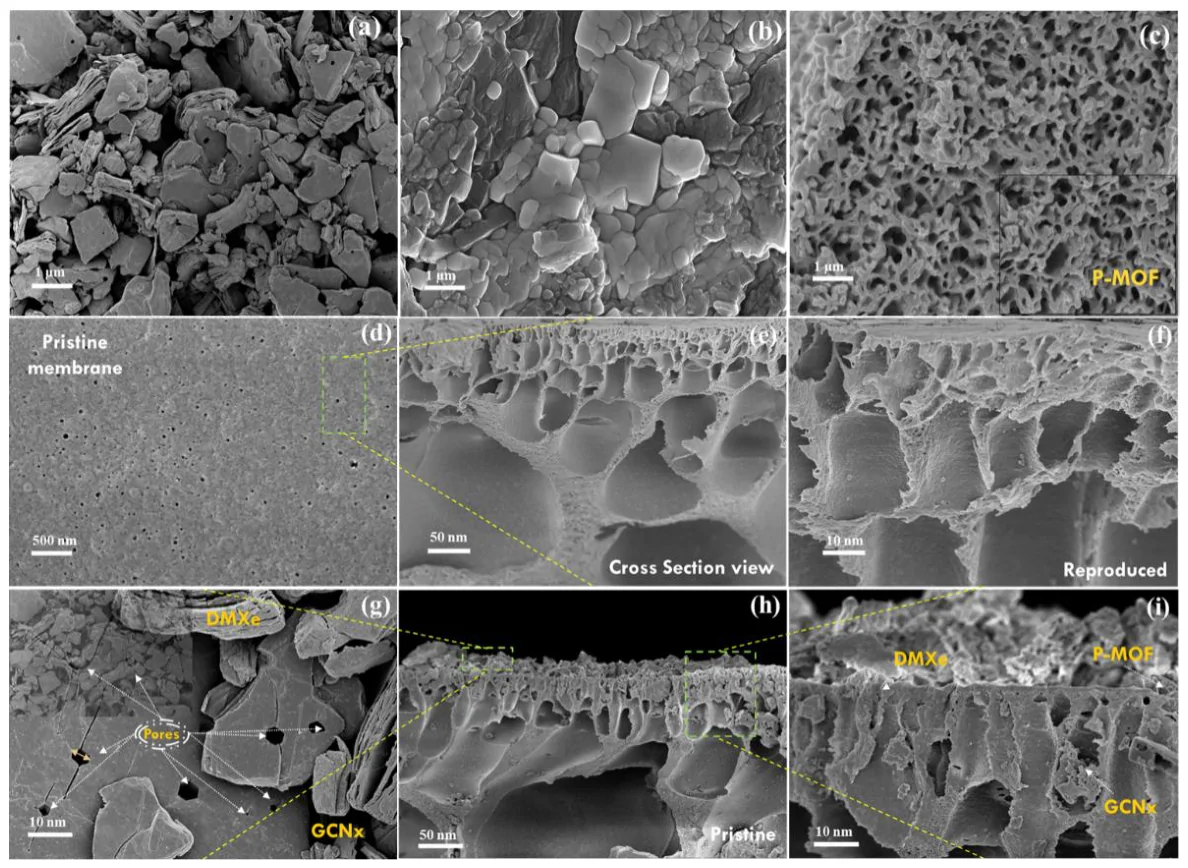
Structural morphology of as-prepared (**a**) DMXe, (**b**) GCN_5_, (**c**) P-MOF, (**d**) Pristine PAN membrane, (**e**) cross-section view, (**f**) reproduced membrane, (**g**) TGCNM (lines indicate porosity), and (**h**,**i**) TGCNM coated membrane (the dashed square denotes the region magnified in the inset image), respectively.

As illustrated in Figure 1a–c, the fabrication of the Ti_3_C_2_@GCN heterojunction commenced with the selective etching of Al layers from Ti_3_AlC_2_ MAX phase precursors. A LiF/HCl mixed solution was employed to remove Al atoms, followed by centrifugal washing to obtain monolayer Ti_3_C_2_ MXene nanosheets [20].

Subsequently, Ti_3_C_2_ MXene/trimesic acid (C_9_H_6_O_6_) and polyvinylpyrrolidone (PVP) were introduced into a suspension. The reducing and coordinating effects of trimesic acid drove the directional assembly of ions with PET ligands on the MXene surface, achieving in-situ epitaxial growth of a Ti-BTC metal–organic framework [21]. The plausible resulting precursor was then subjected to sulfidation in a thioacetamide (TAA)/methanol water mixed system triggered ion exchange and lattice reconstruction within the framework, ultimately forming GCN nanoparticles [22]. Throughout this transformation, the 2D conductive network of MXene remained intact, enabling the construction of a tightly coupled Ti_3_C_2_@ heterointerface. Finally, programmed thermal treatment (heating rate: 2 °C/min^−1^ under N_2_ atmosphere) was conducted at 600 °C for 2 h to optimise the heterointerface. This annealing process promoted lattice mapping and chemical bonding between Ti_3_C_2_ MXene and GCN, yielding the structurally stable Ti_3_C_2_@GCN heterojunction. A systematic investigation of microstructural evolution across Ti_3_C_2_@BTC, intermediate product Ti_3_C_2_@GCN, P-MOF, and final product Ti_3_C_2_@GCN was conducted using scanning electron microscopy (SEM). As illustrated in Figure S2 (SI), the Ti-BTC particles demonstrated uniform dispersion across the Ti_3_C_2_ MXene nanosheet surfaces, exhibiting well-defined particulate morphology with an average particle size of ≈50 nm. Subsequent sulfidation treatment of Ti_3_C_2_@BTC (Figure S3, SI) resulted in the formation of a homogeneous heterojunction interface, where GCN nanoparticles maintained spatial uniformity while achieving intimate contact with the Ti_3_C_2_ MXene substrate. Notably, thermal treatment of GCN at 600 °C (Figure 1d,e) induced significant particle agglomeration, reflecting inherent thermal instability. However, the strategic incorporation of Ti_3_C_2_ MXene as a structural matrix (Figure 1f,g) effectively mitigated this agglomeration phenomenon, facilitating the successful synthesis of thermally stabilised Ti_3_C_2_@GCN heterostructures with preserved interfacial integrity and enhanced morphological regularity (Figure 1h,i). Energy Dispersive Spectroscopy (EDS) elemental mapping (Figure S3) conclusively verified the coexistence of C, N, Ti, and O species within the composite architecture, providing direct chemical evidence for the successful fabrication of Ti_3_C_2_@GCN heterostructures.

**Figure 2:**
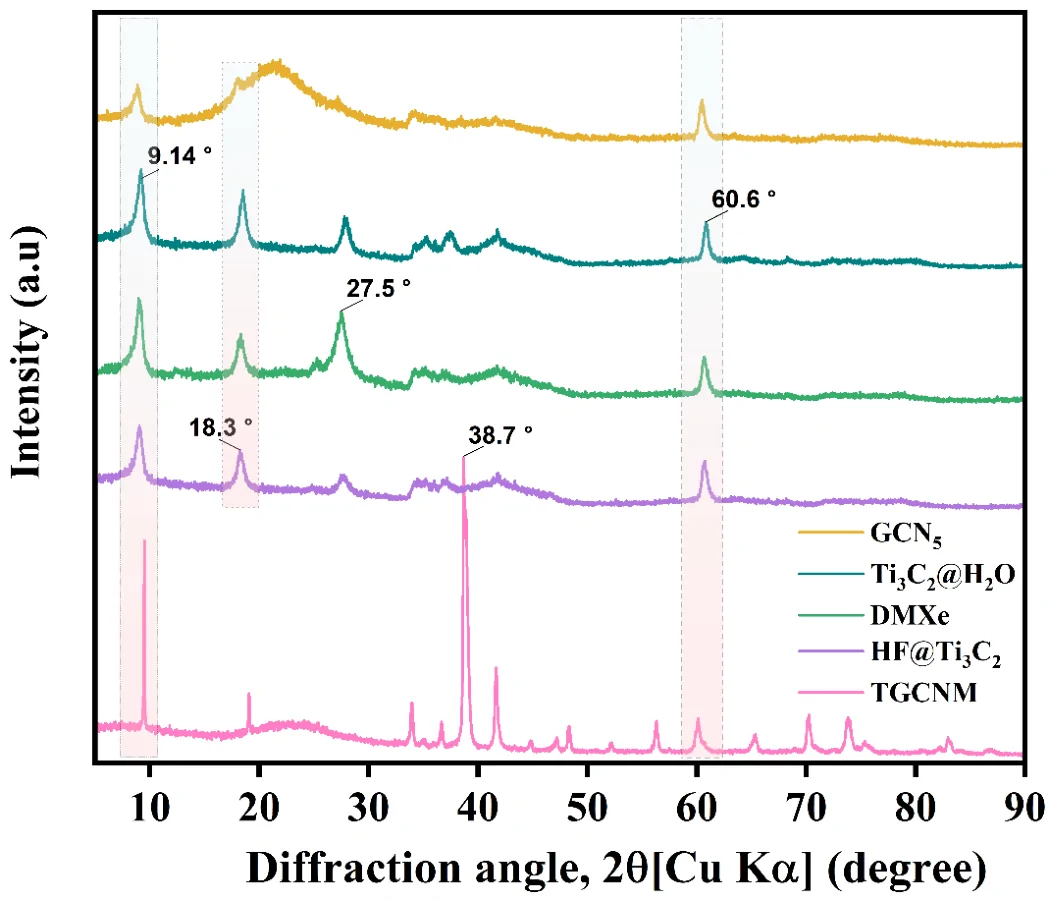
XRD of the used TGCNM photocathode.

Figure 2 presents the X-ray diffraction (XRD) patterns of Ti_3_C_2_@GCN, GCN, Ti_3_C_2_@P-MOF, and TGCNM composites. The characteristic diffraction peaks of TGCNM show excellent consistency with previously reported structural data, confirming the successful fabrication of this composite. The Ti_3_C_2_@GCN composite exhibits distinct diffraction peaks at 29.2°, 31.7°, and 47.7°, which correspond to the (102), (103), and (107) crystallographic planes of hexagonal GCN (JCPDS 87-1526), respectively [23]. This phase-mapping analysis unambiguously demonstrates the effective immobilisation of GCN nanoparticles on the Ti_3_C_2_ MXene surface. Furthermore, three prominent diffraction peaks observed at 32.6°, 39.0°, and 45.4° in the TGCNM pattern are indexed to the (103), (104), and (114) planes of Ti_3_C_2_ (JCPDS 52-0875).

In addition, Figure 2 illustrates the XRD patterns of DMXe, Ti_3_C_2_@H_2_O, Ti_3_C_2_@HF, GCN_5_, and TGCNM. Research using Fourier transform infrared spectroscopy (FTIR) sheds light on how surface functions evolved structurally in all four material systems. Characteristic absorption bands at 3420 and 1640 cm^−1^, persistent in all specimens, are attributed to O–H stretching vibrations of hydroxyl groups and C=O stretching modes inherent to the Ti_3_C_2_ MXene matrix [24]. The Ti_3_C_2_@GCN composite exhibited distinctive vibrational signatures at 760 and 730 cm^−1^, corresponding to aromatic C–H bending vibrations characteristic of the Ti–GCN framework. In addition, the presence of retained coordination environments in the metal organic framework was confirmed by the diagnostic peaks at 1630/1560 cm^−1^ and 1444/1371 cm^−1^, which were attributed to the symmetric and asymmetric stretching vibrations of carboxylate groups (–COO–), respectively [25]. Significantly, the sulfidation-derived materials (Ti_3_C_2_, GCN, and TGCNM) revealed emergent absorption features at 596 and 1120 cm^−1^, corresponding to Ti–O stretching vibrations and characteristic lattice vibrational modes of covellite-phase Ti coordination [26]. Spectroscopic fingerprinting confirmed in-situ Ti–N bonding on Ti_3_C_2_ MXene, retaining vibrational coherence post-high-temperature treatment. Spectral-to-synthetic stage correlations demonstrate controlled phase transformation and interfacial stability in the heterostructure. Furthermore, BET analysis demonstrates that MOF integration significantly enhances the specific surface area of TGCNM (Table S1, SI). With a larger BET specific surface area, TGCNM may be able to adsorb more UO_2_^2+^ than the original GCN.

**Figure 3:**
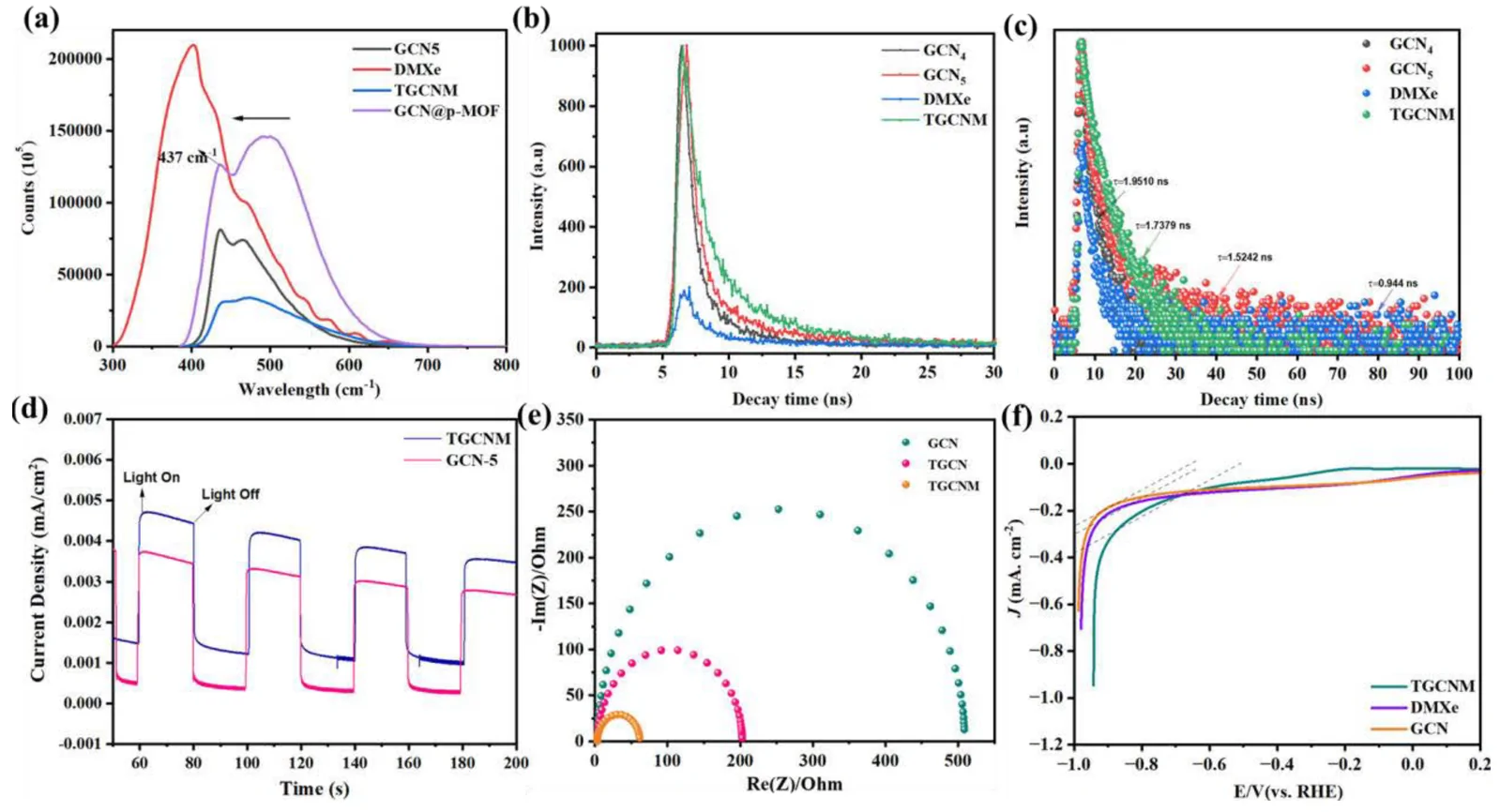
Images of (**a**) photoluminescence (PL) spectra, (**b**,**c**) time-resolved PL, (**d**) transient photo current response, (**e**) EIS, and (**f**) Mott–Schottky flat band potential plot measurement for as-prepared catalysts.

**Figure 4:**
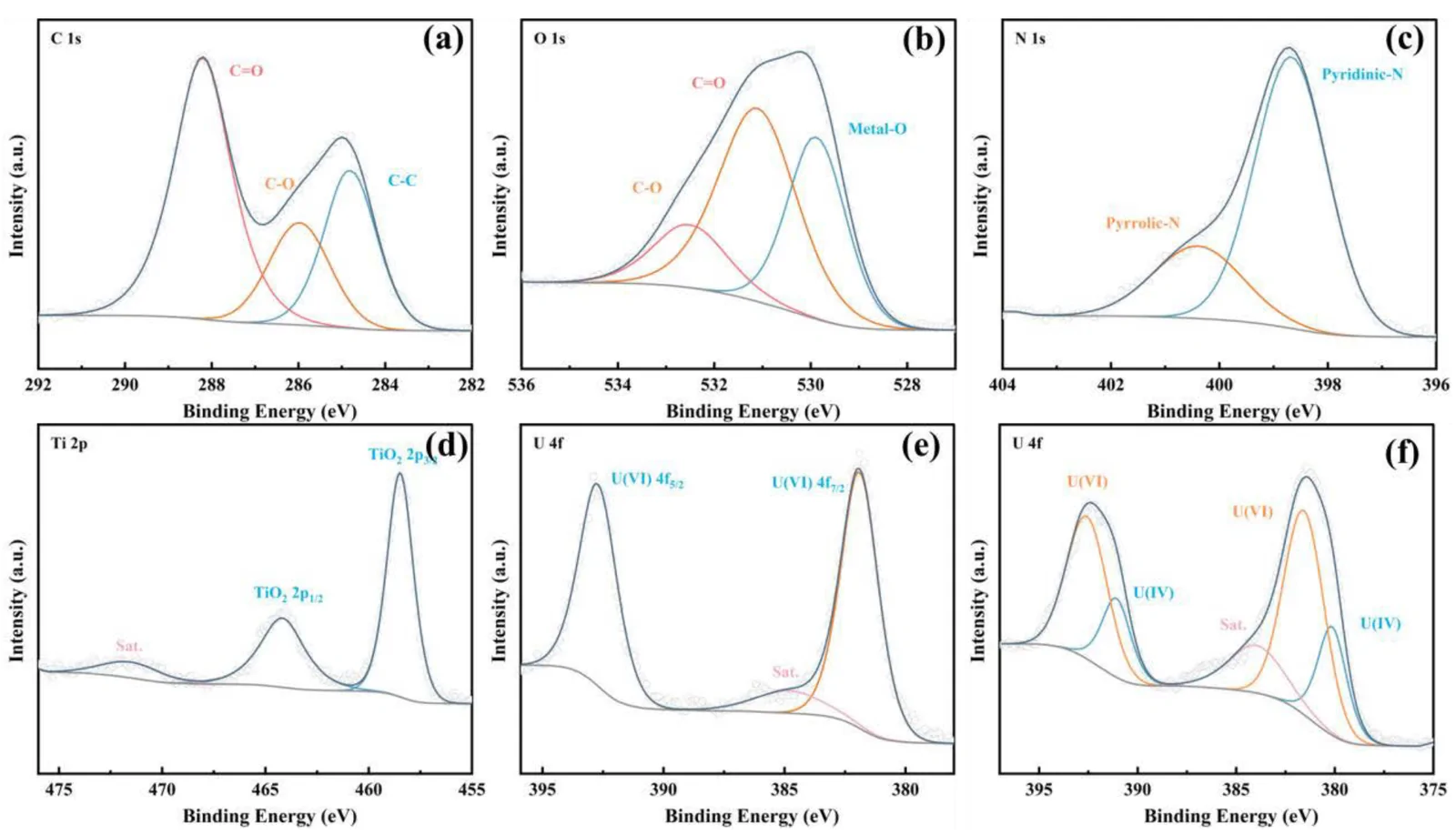
XPS spectra of as-prepared photocathode (**a**) C1s; (**b**) O1s; (**c**) N1s; (**d**) Ti 2p; (**e**) U 4f (under dark); and (**f**) U4f (under AM 1.5 light illumination with 100 W/cm^2^ flux intensity with the lower recombination rate).

The recombination of photogenerated electrons is analysed using the optical studies, which have lower intensity of peak on addition of dopant to the pristine, as shown in Figure 3a–c. As a result of well-planned interfaces, the electrochemical performance of the composite cathode was much improved (Figure 3d). Nyquist plot analysis (Figure 3e) revealed a substantially reduced semicircle diameter for TGCNM compared to pristine CF, indicating decreased charge transfer resistance at the electrode–electrolyte interface. Two complementary elements are responsible for this enhancement: (1) The introduction of Ti–N interfacial bonds allowed for the effective transfer of electrons across the heterojunction. (2) Ionic accessibility was improved throughout the composite design due to the thermally generated mesoporous structure. Cyclic voltammetry measurements (Figure 3f) further confirmed these findings. The electrochemical active area and current density of TGCNM were much greater than CFs at all scan rates. Results from impedance/voltammetric analyses demonstrating that heat processing and MXene GCN integration overcome charge transfer limitations in energy storage confirm that optimising synergistic mass/charge transit enhances electrochemical activity. Carbon/chitin nano film nanorod photoanode morpho-functional analyses and autonomous biasing configurations are detailed in Text S2 (Figures S9–S12, SI). X-ray photoelectron spectroscopy (XPS) was used to extensively investigate the chemical states and elemental composition of TGCNM. As evidenced by the XPS survey spectrum (Figure 4a–d and Figure S3a, SI), the coexistence of N, O, Ti, C, and N elements was confirmed, demonstrating excellent consistency with the elemental mapping results obtained from energy-dispersive spectroscopy (EDS). Deconvolution of the N 1s spectrum (Figure 4c) identified multiple nitrogen-containing species, with binding energies at 396.2 and 401.4 eV assigned to Ti–O–N respectively [27]. This comprehensive analysis provides conclusive evidence for the coexistence of multiple valence states in the composite material. Deconvolution of the O 1s spectrum (Figure S3b, SI) revealed three components at 530.0 eV (Ti–O), 531.4 eV (Ti–O–C), and 532.8 eV (C–Ti–OH), confirming the coexistence of coordinated oxygen environments [28]. The Ti 2p spectrum (Figure S3a, SI) exhibited characteristic peaks at 456.5/460.7 eV (Ti–C) and 458.7/464.4 eV (Ti–O), consistent with the structural features of partially oxidised Ti_3_C_2_ MXene. In the C 1s spectrum (Figure S3b, SI), binding energies at 282.0, 284.7, 286.2, and 287.8 eV were attributed to C–Ti, C–C, C–O, and C–O–Ti bonds, respectively. The increasing oxidation of Ti_3_C_2_ MXene during thermal treatment at 600 °C is the main cause of the observed decline in C–Ti bond intensity. This occurs when oxygen is incorporated, partially converting Ti–C coordination into Ti–O links [29].

### 2.2. ALCE Performance Evaluation

To effectively remediate complex nuclear wastewater matrices produced by industrial operations, co-removal techniques are required because persistent aromatics (phenolic/halogenated chemicals) form redox active complexes with U(VI) [30]. This study utilised bisphenol A (BPA), an antibiotic that is commonly found in water, as a model organic pollutant to assess effectiveness (Figure S13, SI). Cathode optimisation experiments revealed that TGCNM exhibits superior efficiency in concurrent BPA degradation and UO_2_^2+^ removal (Text S7, Figure S14, and Table S2, SI). As illustrated in Figure 3a–d, this study systematically evaluates the synergistic elimination efficacy of BPA and UO_2_^2+^ across multiple treatment systems (complete comparative experimental parameters in Text S8, SI). The carbon/chitin nano film nanotube array (CNF) photoanode showed limited pollutant treatment performance under dark phase adsorption conditions, removing 15.4% of BPA after 40 min and just 14.7% of uranium over 120 min. With the CF cathode, adsorption capacity was much improved, allowing for 28.7% BPA elimination and 24.7% UO_2_^2+^ immobilisation in the same time frames as the other cathode (102/120 min). Most notably, the TGCNM composite cathode demonstrated outstanding dark-phase synergistic ad-sorption performance, with a 50.7% UO_2_^2+^ and 51.4% BPA adsorption rate, which is above the 50% criterion [31]. This can be attributed to the large specific surface area and abundant active sites provided by the MXene framework, as well as the reducing power of S^2−^.

Limited remediation efficiency (<5% pollutant elimination) was observed under standardised air mass (AM) 1.5 irradiation using the standalone photolytic system. Incorporation of CNF photocatalyst marginally improved UO_2_^2+^ elimination (18.3%) and BPA degradation (19.6%) via h^+^-mediated oxidation and e^−^ driven reduction pathways, though overall efficiency remained below 20% due to rapid photogenerated carrier recombination. The ALCE-CF configuration optimised interfacial charge transfer, elevating UO_2_^2+^ removal kinetics to 0.0036 min^−1^ (35.8% efficiency) and BPA degradation rate to 0.013 min^−1^ (41.8% efficiency) (Figure S15). Remarkably, the ALCE-TGCNM cathode-driven system achieved near-complete contaminant eradication (UO_2_^2+^: 96.7%, k = 0.028 min^−1^; BPA: 97.7%, k = 0.087 min^−1^), demonstrating 7.7- and 6.6-fold enhancements in uranium removal kinetics and organic degradation efficiency, respectively, compared to the ALCE-CF. The system demonstrated exceptional photoelectrochemical characteristics with an open-circuit voltage (V_OC_) of 2.38 V and short-circuit current density (J_SC_) of 1.43 mA cm^−2^, achieving a fill factor (FF) of 53.52% and maximum power output (P_max_) of 1.82 mWcm^−2^ (Figure S16; Table S7, SI). This energy conversion capacity enabled commercial light-emitting diode (LED) operation (Figure S16, SI), substantiating its practical applicability. The TGCNM composite combines photo-driven uranyl/organic pollutant reduction with self-sustaining electrocatalytic energy recycling to enable autonomous environmental restoration.

### 2.3. Mechanistic Analytics of ALCE

A thorough investigation was conducted to understand how ALCE removes UO_2_^2+^ and organic pollutants. EDS mapping, XRD, and XPS were all part of the extensive characterisation process. Morphological characterisation revealed disordered fibrous architectures in the TGCNM cathode (Figure S17a,b), with surface-anchored nanoparticles (Figure S17c) potentially corresponding to adsorbed uranium species. Spatial elemental mapping (Figure S17d) and EDS analysis (Figure S17e,f, SI) confirmed homogeneous U distribution (48.8%) across the TGCNM cathode surface, demonstrating effective U enrichment at the ALCE-TGCNM interface. XPS deconvolution of U 4f spectra (Figure 3e) quantified U speciation as 83.0% U(IV) and 12.5% U(VI), indicating predominant reductive removal over adsorptive retention [32]. In adsorption systems, the increased U signal strength on ALCE-TGCNM in comparison to Dark-TGCNM further confirmed this distribution pattern (Figure 4f). Complementary XRD analysis (Figure S17, SI) exhibited characteristic UO_2_ diffraction peaks, confirming crystalline uraninite as the dominant reduction product.

The underlying signals in the 396–405 eV range are consistent with reference binding energies of pyridinic-N, pyrrolic-N, graphitic-N, and oxidised-N components for g-C_3_N_4_. High-resolution N 1s spectroscopy (Figure 4c) reveals an emerging nitrogen signature at 169.9 eV with a concomitant decrease in S^2−^ intensity. The sequence of this oxygen species proves the gradual decline of the oxygen in the process of supplying electrons to uranium (VI) reduction [33]. XPS analysis indicates that Dark-TGCNM spontaneously reduces in the dark, as indicated by reduced UO and increased NUO. The intrinsic driving force for electron donation by S^2−^ primarily stems from the strong binding interactions between S^2−^ and UO_2_^2+^, which facilitate the reduction of U(VI). Furthermore, low-valent metal cations have the potential to facilitate redox reaction, through which higher separation and transfer of photoexcited electrons are involved in the photoreduction of uranium ions in seawater. The reduction of high-valent metal ions to their lower-valent states is facilitated by this synergistic mechanism, which also speeds up the kinetics of electron transfer. The oxygen coordination environment evolution was evidenced by new U=O bonding features at 31.0 eV in O 1s spectra (Figure S18, SI), corroborating uranium oxide formation. At the same time, U-species surface coordination is likely to blame for the reduced C–Ti–OH component. Surface sites may be obscured by deposited uranium species, which could explain the attenuation of the Ti 2p spectrum intensity (Figure S18, SI). Notably, the C1s spectrum remained essentially invariant across treatments, confirming structural stability of the carbon framework during redox processes [34].

**Figure 5:**
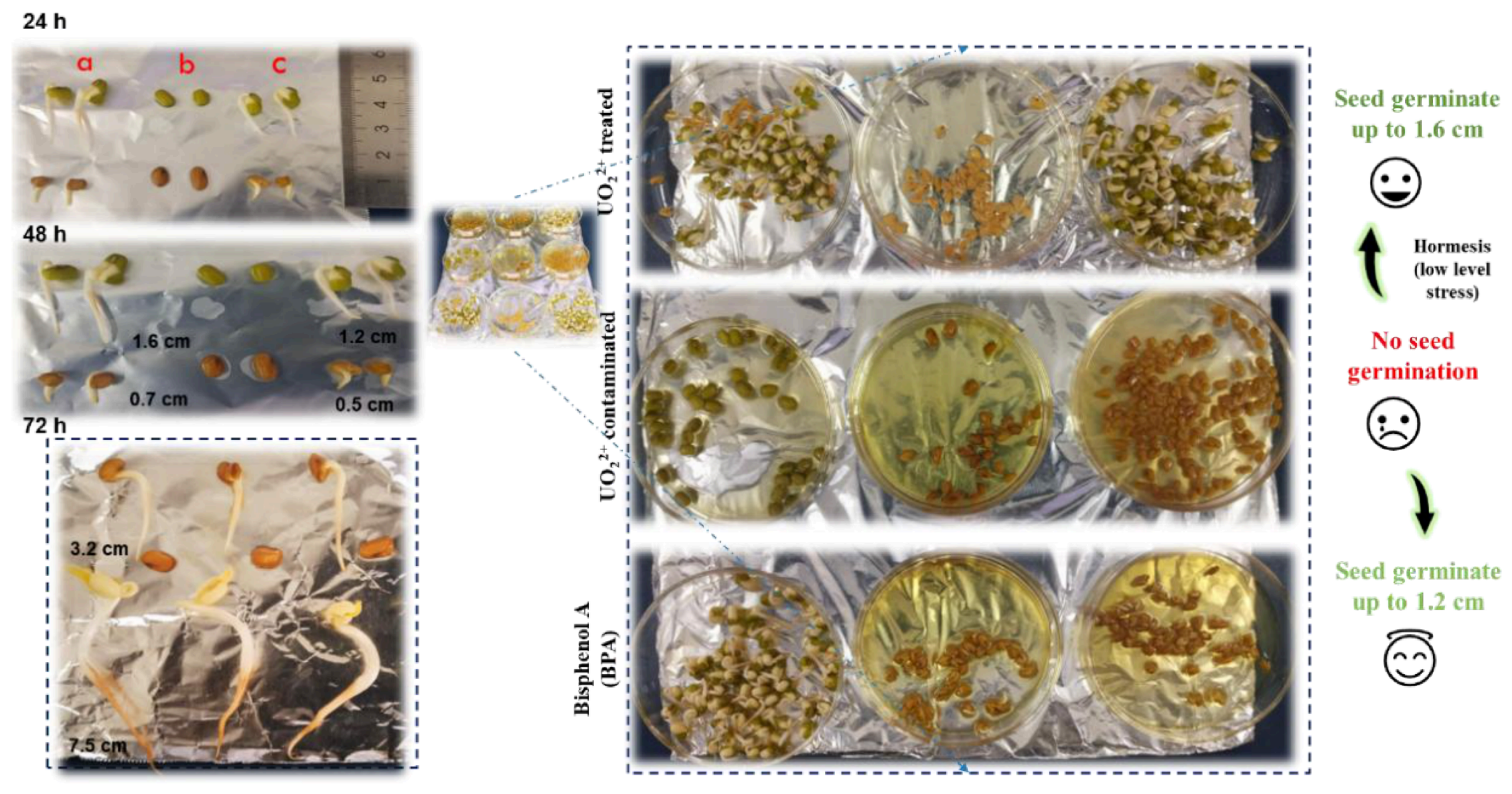
Toxicity evaluation of treated UO_2_^2+^ and BPA eluent on seed germination and root elongation with the higher catalytic removal (inset shows the various size formation of the root on toxicity evaluation with the higher concentration of BPA on toxic environment).

Figure 4 and Figure 5 illustrate the possible synergistic decontamination mechanism of the ALCE-TGCNM system toward U(VI)BPA co-pollutants. Text S9 and Figures S19–S21 (SI) analyse the degradation pathway and toxicity analysis of BPA. Under solar illumination, the CNF photoanode absorbs short-wavelength photons (*λ* < 412 nm) to generate e^−^/h^+^ (Equation (1)) [17]. Photogenerated holes migrating to the surface oxidise adsorbed H_2_O molecules into •OH radicals (Equation (2), Figure S22, SI). These radicals collaborate with holes to achieve complete BPA degradation via non-selective oxidation (Equations (3) and (4)) [35]. In addition, EPR and UV–vis analyses confirmed the absence of detectable •O^2−^ or H_2_O_2_ during U(VI) removal (Figure S23, SI). Quantitative comparison of EPR signal intensity across scavenger conditions indicates that superoxide radicals (•O_2_^−^) and photogenerated holes (h^+^) are the dominant reactive species, with hydroxyl radicals (•OH) playing a secondary role, consistent with the relative suppression of BPA degradation observed upon introduction of the corresponding radical scavengers (p-benzoquinone, triethanolamine, and isopropanol, respectively). The Si PVC back electrode establishes a self-biased electric field by harvesting long-wavelength photons (Equation (5)), which drives photo-electrons toward the highly conductive Ti_3_C_2_@GCN_5_ cathode (Equation (6)), as evidenced by LED photo response experiments (Figure S15, SI). The negatively charged functional groups on Ti_3_C_2_ MXene enable efficient UO_2_^2+^ adsorption via electrostatic interactions (Equation (7)), while the interfacial Ti–O–C bonds enhance charge separation through dynamic redistribution mechanisms, facilitating stepwise reduction of UO_2_^2+^ to UO_2_ precipitates (Equation (8)). Simultaneously, Ti^2+^ in the Ti_3_C_2_ lattice function as co-electron donors. These species undergo partial oxidation to Ti^2+^ (Equations (1)–(8)), thereby stabilising U(IV) species through a redox-coupled pathway. It is worth noting that electrons generated by the CNF anode dominate the UO_2_^2+^ reduction process, enabling the ALCE composite material to maintain a removal efficiency of > 96% even at a concentration of 200 mg L^−1^ UO_2_ ^2+^ (Figure S24, SI).

(1) $$h^{+}+H_{2}O\to \;•OH$$

(2) $$h^{+}+BPA\to \;H_{2}O+{CO}_{2}$$

(3) $$•OH+BPA\to \;H_{2}O+{CO}_{2}$$

Si PVC + hν (λ = 400–600 nm) → Bias voltage (4)

(5) $$e^{-}\left(CNF\right)\to e^{-}(TGCNM)+electricity$$

(6) $${UO}_{2}^{2+}+TGCNM\to {\left({UO}_{2}^{2+}\right)}_{ads}+\left(\left(TGCNM\right)\right)\;{\left({UO}_{2}^{2+}\right)}_{ads}+\left(\left(TGCNM\right)\right)+{2e}^{-}\left(TGCNM\right)$$

(7) $$\to {UO}_{2}\left(S\right)+\left(\left(TGCNM\right)\right)$$

(8) $${UO}_{2}^{2+}+H_{2}O\to {UO}_{2}\left(S\right)+4{OH}^{-}$$

### 2.4. Adaptation and Application Potential of ALCE

**Figure 6:**
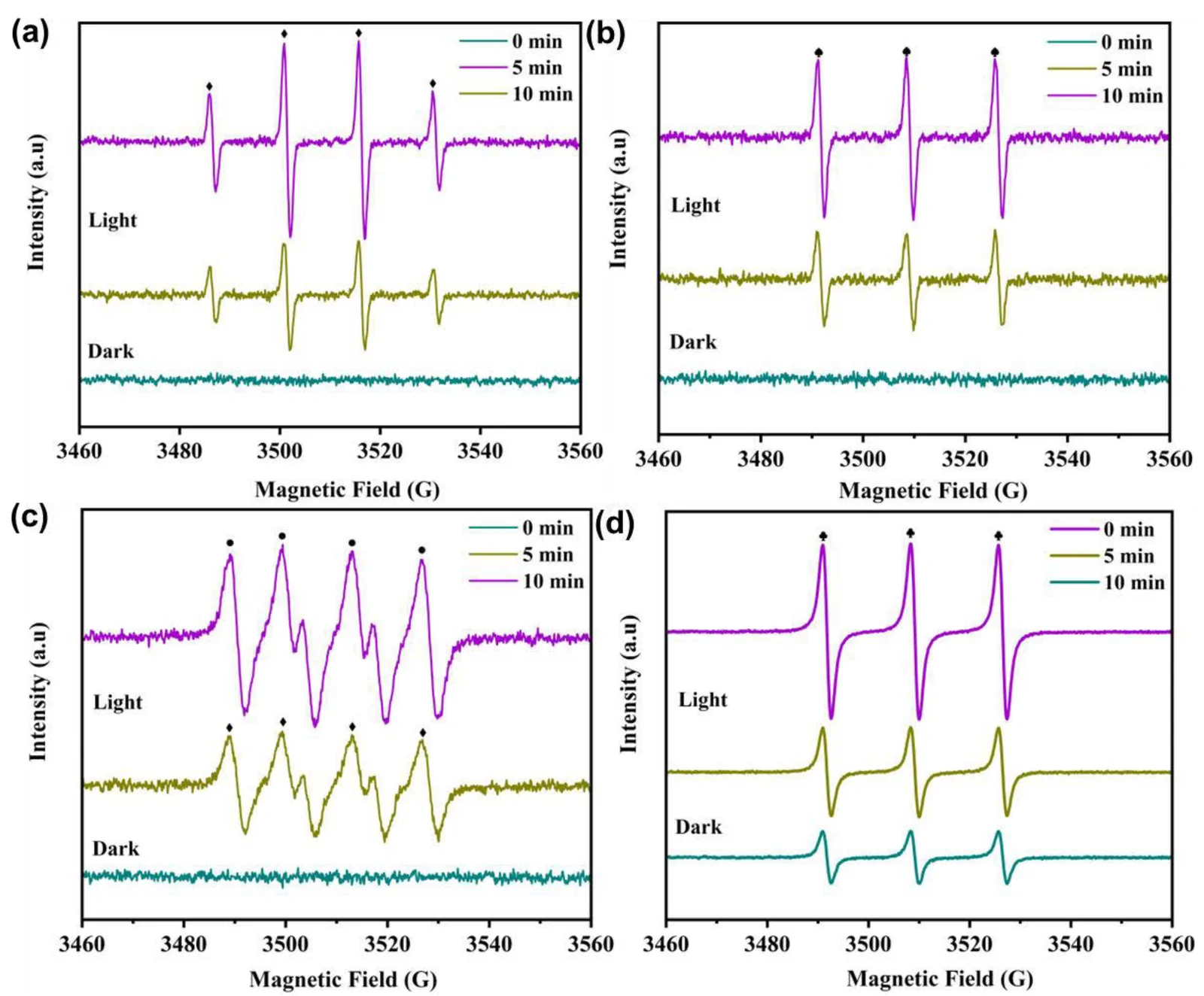
Scavenging radical experiment (**a**) •OH, (**b**) •O_2_^−^, (**c**) h^+^, and (**d**) _1_O^2^ radical, respectively on light irradiation of TGCNM with operational parameters. (Operational parameters such as temperature = 27 °C; dosage = 30 mg/L; initial concentration = 10 mg/L; light intensity = AM 1.5 100 mW/cm^2^). EPR spectra were recorded in the dark and under light irradiation at 0, 5 and 10 min. The symbols (◆, ♠, ●, ♣) mark the characteristic signals of the trapped radical adducts in each panel: (**a**) [DMPO-•OH], (**b**) [DMPO-•O_2_^−^], (**c**) [DMPO-h^+^ adduct], and (**d**) [TEMP-^1^O_2_]. Spin-trapping agents: [DMPO for (**a**–**c**) and TEMP for (**d**)].

Efficient reduction of UO_2_^2+^ and degradation of BPA are determined by wastewater conductivity, which in turn controls the flow of electrons between the CNF and the cathode by adjusting the internal resistance of the ALCE [36]. Figure 6a illustrates the performance of ALCE under varying sodium sulfate concentrations (0, 0.02, 0.05, 0.10, and 0.5 m). In the absence of electrolyte (Na_2_SO_4_), the removal efficiencies of UO_2_^2+^ and the BPA were limited to 53.57% and 68.12%, respectively, with a P_max_ of 0.05 mW cm^−2^ (Figure S25 and Table S4, SI). When Na_2_SO_4_ concentration increased to 0.5 m, the removal efficiencies of U(VI) and BPA reached 99.65% and 100%, accompanied by a J_SC_ elevation to 1.72 mA cm^−2^.” This enhancement can be attributed to improved solution conductivity at higher Na_2_SO_4_ concentrations, which effectively reduces internal resistance and facilitates electron transfer, thereby promoting UO_2_^2+^ reduction, BPA degradation, and power generation. The 0.1 M Na_2_SO_4_ supporting electrolyte was included to increase solution ionic conductivity, thereby reducing ohmic resistance and improving charge-transfer efficiency across the electrode–electrolyte interface. In its absence, the intrinsically low ionic strength of the synthetic wastewater would be expected to increase internal resistance and correspondingly reduce photocurrent and removal efficiency, consistent with standard photoelectrocatalytic cell operation. After careful consideration of removal efficiency and cost-effectiveness, the ideal electrolyte concentration for the following tests was determined to be 0.1 m Na_2_SO_4_. We further evaluated ALCE removal efficiency for UO_2_^2+^ and BPA under varying irradiance levels (Figure S26, SI). Both species were completely eliminated (100%) at irradiances greater than 200 mW cm^−2^. “This reveals that ALCE is much more effective at removing UO_2_^2+^ and BPA when using increased light harvesting. Also, by influencing its chemical form, solution pH controls uranium’s photoreduction pathway. As shown in Figure 6b, the ALCE achieved peak UO_2_^2+^ removal efficiency (96.73%) at pH 5.0, with a sharp decline to 44.2% as pH decreased to 2.0. At the TGCNM cathode, where an abundance of H^+^ ions impeded uranyl ion reduction, competitive proton–electron interactions caused this degradation. Conversely, alkaline conditions (pH 5.0–8.0) induced surface charge inversion through hydrolysed uranyl complexes [(UO_2_)_3_(OH)_5_^+^, (UO_2_)_4_(OH)_7_^+^] [37], creating electrostatic repulsion that reduced reduction kinetics to 85.56% at pH 8.0. Kinetic data for both U(VI) removal and BPA degradation are best described by pseudo-first-order behaviour, consistent with the Langmuir–Hinshelwood kinetic framework and consistent with a surface-adsorption-mediated, rate-limiting reaction step at the concentrations tested, as tabulated in Table S15. Remarkably, the system maintained practical operability with >85% uranium removal across this pH spectrum, and the pH value changed relatively little after the reaction (Figure S27, SI), with only slight fluctuations. Primarily driven by hole-mediated oxidation at CNF photoanodes and hydroxyl radical chain reactions, the BPA degradation efficiency stayed consistently high (>92.7%) throughout pH 2–8. The ALCE exhibited stable power output parameters: J_SC_ (1.43–1.78 mA cm^−2^), V_OC_ (2.33–2.50 V), P_max_ (1.43–1.82 mW cm^−2^), and FF (35.66–53.52%) (Figure S28 and Table S5, SI). These results demonstrate robust pH adaptability for treating uranium–organic wastewater through synergistic contaminant removal and bioenergy recovery. The interference of the anion and cation during the removal of uranium and BPA causes resistance over the ALCE system. The maximum ability of interfering ions has the ability to remove the partition on both the pollutants with the different mechanisms. The major mechanisms involved are synergy, electrostatic energy, and higher π–π interaction towards the TGCNM surface interface. The saturation of ions through the maximum interference of both the pollutant and catalyst involves a chemical reaction via the photo-electrochemical process, which clearly induces the formation of the removal process in the ALCE system. The excess ions with the higher removal confirms that the role of competing ions makes the photo-electrocatalytic process more reluctant towards the ALCE system under the AM 1.5 illumination. This concludes the major role of ions involved in the reduction, which promotes photogenerated electrons (e^−^) and holes (h^+^), respectively, for the process of both oxidation and reduction [38]. The removal process in AM 1.5 ALCE illumination has the capacity for reusability in the removal of BPA/UO_2_^2+^.

**Scheme 1:**
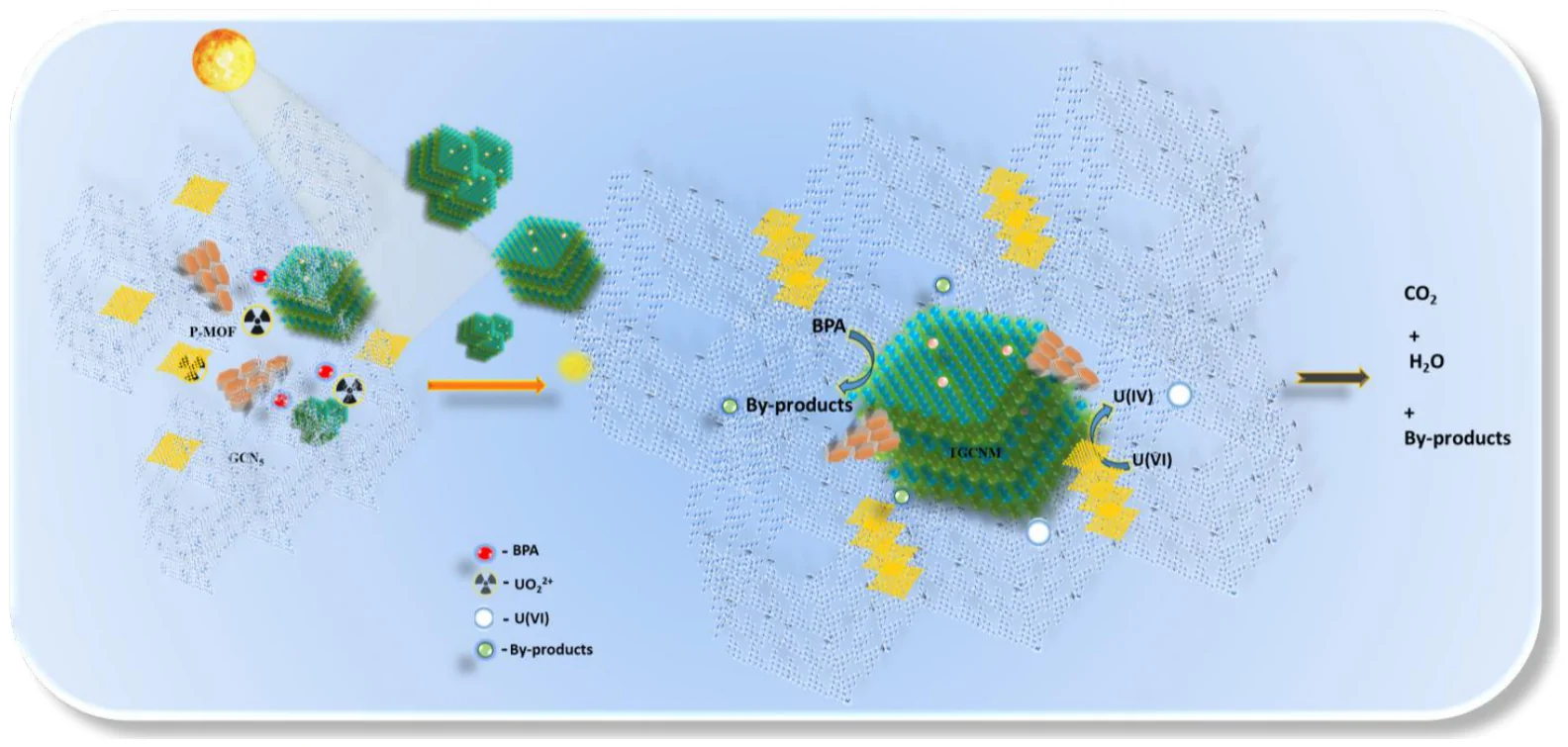
Pictorial representation of proposed mechanism of charge transfer and reaction pathway on BPA and UO_2_^2+^ degradation under visible light illumination via ALCE system (inset shows that the maximum removal capacity on excess of BPA/UO_2_^2+^ configuration).

Using a germination test on mung bean seedlings, we were able to compare the phytotoxicity of untreated and treated water systems that included UO_2_^2+^ and BPA. After seeds were exposed to various water systems, the toxicity study measured the percentage of germination, root length, and stem length (Figure S9). The contaminated and treated water systems were compared to DI, which served as a control. At the 5-day mark of plantation, the seed germination percentage was calculated to be 100% for the control group, 50% for the group exposed to polluted water, and 90% for the group treated with ALCE. (Table S6; Figure S13). Root lengths averaged 1.99 ± 0.80 cm and stem lengths 1.70 ± 0.35 cm in the control system. Uranium, on the other hand, reduced the growth of both the stem and the roots by 0.94 ± 0.21 cm and 2.86 ± 0.79 cm, respectively. Interestingly, the percentage of seed germination, stem length, and root length for the water treated with BPA (1.31 ± 0.24 cm and 3.41 ± 1.68 cm, respectively) was more similar to what was seen in the control system. This indicates that the ALCE under AM 1.5 light was able to breakdown BPA and its intermediates. Degradation of the intermediate and defluoridation analysis further lend credence to this finding. This study proves that the AM 1.5 ALCE system is highly effective at reducing U(VI) and BPA oxidation in water-based matrices as depicted in Scheme 1.

This study established a U(VI)-bisphenol A binary system (UO_2_^2+^: 10 mg L^−1^, BPA: 0–40 mg L^−1^) to elucidate concentration dependent interaction mechanisms (Figure S22, SI). The removal effectiveness of U(VI) from 98.2% to 81.2% was caused by a gradual decrease in BPA concentration (5 → 40 mg L^−1^), which was attributed to competitive adsorption between bisphenol A and uranyl complexes (Figure S22, SI). The system’s remarkable anti-interference capacity was on full display at 40 mg L^−1^ BPA, where it achieved 81.4% uranium elimination and 96.5% antibiotic degradation, notably maintaining robust performance. On the other hand, studies using a constant BPA concentration (10 mg L^−1^) showed that changing the U(VI) concentration (0–40 mg L^−1^) had a negligible effect on co-contaminant removal efficiency (sustained > 92.4%), confirming a high level of operational adaptability (Figure S22, SI). Notably, when the UO_2_^2+^ concentration was reduced to 0.05 mg L^−1^, the removal efficiency of UO_2_^2+^ reached 70.4% (residual concentration reduced to 14.8 μg·L^−1^), which is below the World Health Organization (WHO) standard limit of 30 μg·L^−1^ for uranium content in drinking water. Synchronous electrochemical monitoring revealed minimal energy output fluctuations (V_OC_: 2.25 2.43 V; P_max_: 1.33–1.82 mW cm^−2^) under varying pollutant loads (Figures S23 and S24, Tables S8 and S9, SI). The electricity generation (energy harvesting) process has the tendency to procure LED enlightening with the higher removal tendency of BPA and UO_2_^2+^ species under simulated natural solar light and an artificial xenon lamp with an intensity of AM 1.5 illumination (100 W/cm^−2^).

This dual functional system is a new option for complex wastewater treatment scenarios due to the demonstrated synergy between pollutant remediation efficacy and steady bioenergy recovery. The coexistence of multivalent metals (e.g., Cu^2+^/Cd^2+^) with competing anions (e.g., Br^−^/NO_3_^−^) in industrial wastewater significantly inhibits pollutant removal. With the use of simulated wastewater (anion: 10 mmol L^−1^; cation: 2 mmol L^−1^), this study examined the methods for removing pollutants that are specific to certain ions. As illustrated in Figure 6c, the coexistence of chloride (Cl^−^) and bromide (Br^−^) ions markedly enhanced both UO_2_^2+^ removal and BPA degradation efficiencies. This phenomenon can be attributed to the generation of chlorine (Cl^−^) and bromine (Br^−^) radicals through hole (h^+^) scavenging by Cl^−^ and Br^−^, which effectively degraded BPA molecules and disrupted their complexation with UO_2_^2+^. Reactive halogen species (e.g., Cl•, Br•, and related radicals) generated in the presence of Br^−^/Cl^−^ may contribute to the formation of halogenated organic intermediates from phenolic parent compounds such as bisphenol A, consistent with mechanisms documented in the report of water-treatment survey. This represents an important consideration for deployment of this system in halide-rich wastewater matrices; halide-content screening and, where necessary, an extended treatment duration or polishing step are recommended to minimise the potential for transient halogenated by-product accumulation as tabulated in Table S10. Notably, both dihydrogen phosphate (H_2_PO_4_^−^) and bicarbonate (HCO_3_^−^) anions demonstrated exceptional UO_2_^2+^ removal efficiencies exceeding 95%, while HCO_3_^−^ exhibited a relatively minor impact on BPA degradation (88.1%). The reduced BPA degradation efficiency in the presence of HCO_3_^−^ may result from its scavenging effect on hydroxyl radicals (•OH), which are crucial oxidative species in the degradation process. In contrast, nitrate (NO_3_^−^) induced a marked decrease in UO_2_^2+^ removal performance, potentially due to its weak complexation affinity with (UO_2_)_3_(OH)_5_^+^ species compared to other anions, thereby limiting co-precipitation effectiveness.

Furthermore, transition metals (Ca^2+^, Mg^2+^, Zn^2+^, Ni^2+^, Cd^2+^, Cu^2+^, and Fe^3+^) exhibited competitive interfacial effects, driving ALCE’s cation resistance evaluation. As depicted in Figure 6d, Fe^3+^ and Cu^2+^ caused substantial declines in UO_2_^2+^ removal efficiency to 62.0% and 65.8%, respectively. This inhibitory effect stems from their elevated redox potentials (E°(Fe^3+^/Fe^2+^) = 0.77 V vs. SHE; E°(Cu^2+^/Cu) = 0.34 V vs. SHE), enabling competitive electron-accepting behaviour that diminishes electron availability for UO_2_^2+^ reduction (E°(UO_2_^2+^/UO_2_) = 0.411 V vs. SHE). Herein, the higher removal of BPA and uranium(U(VI)) on complex waters shows excellent contrast. Other cations exhibited negligible interference (<5% efficiency loss), likely due to their lower reduction potentials (E°(M^2+^/M) < 0 V vs. SHE) that disfavoured electron competition [39]. Remarkably, BPA degradation maintained >96% efficiency across all cationic systems, driven primarily by hole-mediated oxidation rather than reductive pathways. While BPA parent-compound removal reached 97.7%, the degradation pathway is expected to proceed through smaller, more oxidised intermediate species (e.g., hydroquinone- and catechol-type structures, consistent with reported BPA advanced-oxidation degradation pathways) prior to eventual mineralisation to CO_2_ and H_2_O. This is consistent with the removal rate for advanced-oxidation-based degradation of bisphenol A, with a removal rate of 97.7 ± 0.8 on HPLC (Figure S5) and 68.4% of TOC. COD with a 72.6% mineralisation extent is expected to be somewhat lower than parent-compound removal, indicating substantial but not complete mineralisation. The photoelectrochemical property of the cathode has a higher removal rate with the passage of electrons on the electrochemical than the photocathode and anode with the electrochemical performance.

**Figure 7:**
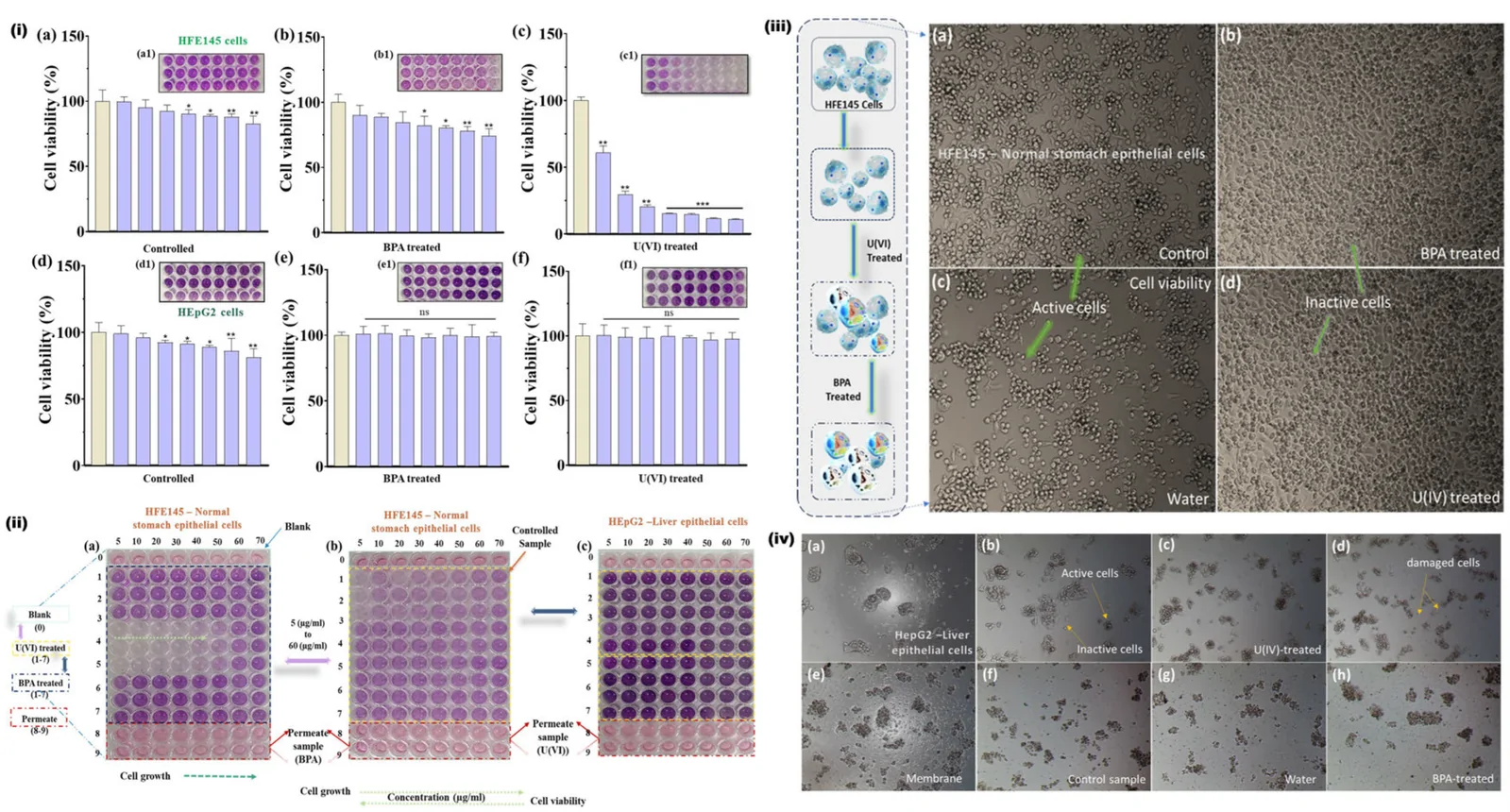
In vitro cytotoxicity and cell proliferation assessment of BPA and U(VI) solutions. (**i**) MTT based cell viability of untreated control cells and cells exposed to BPA and U(VI): (**a**) Controlled; (**b**) BPA treated; (**c**) U(VI) treated of HFE145 cells; (**d**) Controlled; (**e**) BPA treated; (**f**) U(VI) treated of HepG2 cells. Insets (**a1**–**f1**) show the corresponding MTT-stained 96-well plates, in which the purple color intensity is proportional to the number of viable cells. Data are presented as mean ± SD (*n* = 3); * *p* < 0.05, ** *p* < 0.01, *** *p* < 0.001 compared with the untreated control; ns = not significant. (**ii**) Correlation between MTT absorbance and cell number at different sample concentrations: (**a**) Blank; (**b**,**c**) Controlled cell samples. (**iii**) Microscopic examination of cells after treatment, showing no significant cell death: (**a**) Control; (**b**) BPA treated; (**c**) Water; (**d**) U(VI) treated. (**iv**) Viability of HepG2 liver epithelial cells and HFE145 normal stomach epithelial cells treated with BPA and U(VI) solution, assessed by MTT assay, trypan blue exclusion and propidium iodide staining: (**a**) HFE145 cells; (**b**) HepG2 cells; (**c**) U(VI) treated; (**d**) Damaged cells; (**e**) Membrane; (**f**) Control sample; (**g**) Water; (**h**) BPA treated.

Cell proliferation was evaluated to assess the biocompatibility of the treatment process, comparing untreated control cells against cells exposed to BPA and U(VI) individually. The MTT (3-(4,5-dimethythiazol-2-yl)-2,5-diphenyltetrazolium bromide) reduction assay is a widely utilized colorimetric method for assessing in vitro cytotoxicity and cellular proliferation due to its simplicity and sensitivity. As shown in as shown in Figure 7(ia–f), the control group showed the highest cell viability among all groups. BPA exposure reduced cell viability relative to the control, consistent with its known cytotoxic effects, and U(VI) treatment further affected proliferation, reflecting the toxicity associated with residual uranium. In contrast, showed lower viability, indicating reduced cytotoxic impact following treatment. These results support the biocompatibility considerations relevant to the treatment approach developed in this study. It has been shown to correlate effectively with cell number within the limits of absorbance measurement as depicted in Figure 7(iia–c). Therefore, we hypothesize that the various concentration of sample which interferes with MTT reduction without affecting cellular viability. Accurate determination of cell viability is crucial for evaluating the cytotoxic potential of chemical pollutants. Microscopic examination of the cells also did not reveal significant cell death Figure 7(iiia–d). Additionally, it is important to exclude the possibility of extracellular compounds reducing MTT during the assay. In our study, treatment of HepG2 liver epithelial cells and HFE145 normal stomach epithelial cells with BPA and U(VI) solution led to a decrease in cell viability as measured by the MTT assay (Figure 7(iva–h)). Interestingly, we did not observe a corresponding decrease in cell proliferation or survival when assessed using trypan blue exclusion and propidium iodide staining.

**Figure 8:**
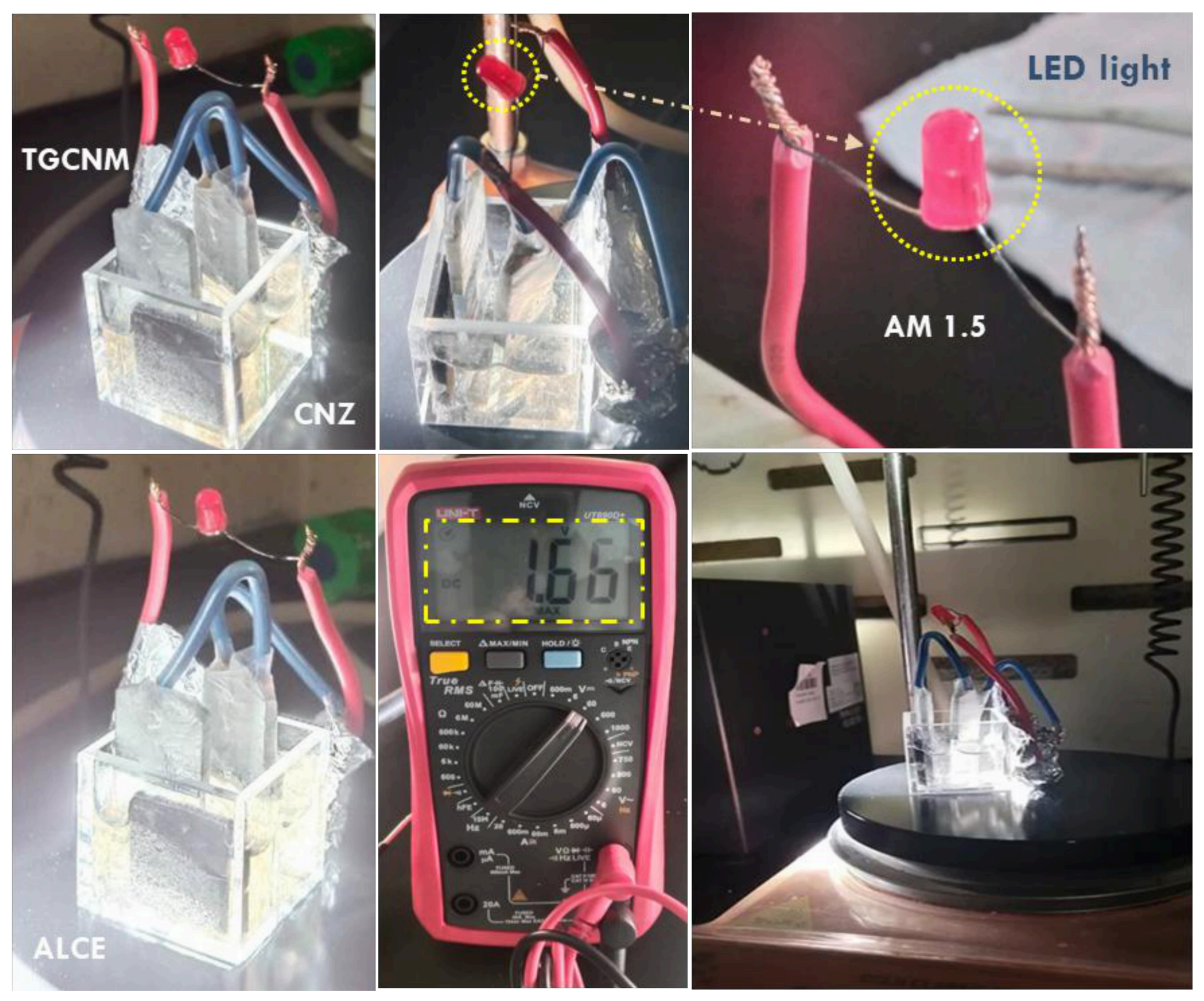
Photographic images depicting the self-driven PEC system in operation under simulated solar irradiation with a LED lamp (inset shows the LED light illumination with the higher current density and potential as highlighted in the yellow dotted lines).

Concurrently, ALCE exhibited stable photoelectrochemical output under multi-ionic conditions (Figures S14 and S15, Tables S10 and S11, SI), demonstrating consistent operational parameters: short-circuit J_SC_ = 1.34–1.66 mA cm^−2^, V_OC_ = 2.36–2.47 V, P_max_ = 1.30–1.82 mW·cm^−2^, and FF = 38.8–53.52%. A multi-metallic synergistic photo-electrocatalytic system for industrial wastewater can be designed on the theoretical basis of the revealed electron transfer mechanism and potential control selectivity [40]. After five iterations of regeneration, the ALCE-TGCNM system’s cyclic stability was systematically assessed (Figure S16). Following each adsorption phase, a 0.1 m sodium bicarbonate eluent effectively stripped the immobilised UO_2_^2+^ complexes from the TGCNM cathode surface, achieving near-complete regeneration of active sites (2UO_2_ + 6CO_3_ ^2−^ + O_2_ + 2H_2_O → 2UO_2_(CO_3_)_3_ ^4−^ + 4OH^−^). Throughout all cycles, the system consistently demonstrated 93.3% uranium retention and 94.3% BPA degradation rates, while exhibiting less than 4.3% activity attenuation in the casing. Furthermore, ALCE’s elution efficiency stayed above 87.6% after five cycles, suggesting strong recyclability (Figure S17, SI). A reduction in the Ti–C ratio relative to the original material was seen in XPS examination of TGCNM/CF after five regeneration cycles; this was explained as oxidation produced by ALCE (Figure S18, SI). Remarkably, the dependability of ALCE was confirmed, since all other XPS peaks stayed the same. This remarkable regeneration capability reduces the amount of consumable materials needed, which is an important operational characteristic that makes photocatalytic nuclear effluent treatment systems more economically viable to install on an industrial scale [41]. The complex chemical makeup of aquatic habitats means that there are now no cost-effective or scalable extraction technologies for uranium that is abundant in seawater [42]. We methodically assessed ALCE’s uranium extraction efficacy in a simulated saltwater matrix that incorporates various co-existing ionic species, building on its high efficacy validation (Table S10, SI). As illustrated in Figure S19, ALCE reached removal rates of about ≈98.4% for BPA and 96.2% for U(VI) resulting from combined functional mechanisms. At the same time, ALCE also demonstrated excellent performance in real seawater and industrial wastewater (Figure S20, SI). Notably, the material concurrently exhibited exceptional electricity generation performance, delivering a P_max_ of 1.54 mW·cm^−2^, FF of 41.21%, J_SC_ of 1.49 mA cm^−2^ and V_OC_ of 2.47 V under standard operating parameters (Figure S21 and Table S11, SI). These multi-functionalities position ALCE-TGCNM as a compelling material for both uranium extraction and harvesting energy from marine environments as depicted in Figure 8.

In addition, ALCE demonstrated versatile remediation capabilities for radioactive wastewater with combined uranium and organic contamination. As evidenced in Figure S14 (SI), this system achieves simultaneous uranium extraction (UO_2_^2+^ removal > 90.3%) and multi-contaminant degradation (>92.4% elimination of ibuprofen (IBU), p-nitrophenol (PNP), atrazine (ATZ), ciprofloxacin (CIP), and sulfamethoxazole (SMZ)). Meanwhile, the configuration of ALCE-TGCNM maintains a stable electrochemical output (Figure S22 and Table S12, SI). This research lends credence to a new way of thinking about the management of nuclear effluent as a kind of engineered treatment for organo–uranium composite pollution. The efficacy of decontamination of hexavalent uranium (U(VI)) is greatly reduced by radioactive wastewater that contains tannic acid (TA) and ethylenediaminetetraacetic acid (EDTA) due to coordination competition effects. As demonstrated in Figure S15a (SI), elevating EDTA concentration from 5 to 50 mg·L^−1^ reduces UO_2_^2+^ removal sequestration from 97.8% to 89.8%, accompanied by a 37.5% decrease in kinetic rate constant (k: 0.023→0.016 min^−1^; Figure S15b, SI). This inhibitory behaviour originates from EDTA’s hexadentate coordination configuration with UO_2_^2+^ (Figure S16, SI), which stabilises uranyl complexes and impedes the interfacial reduction.” In the same way, increasing TA concentration to 50 mg L^−1^ (Figure S17a,b, SI) decreases uranium removal by 3.1% (96.8% → 93.7%) and reduces the rate kinetic constant by 19.5% (k: 0.042 → 0.034 min^−1^), attributable to strong bidentate coordination through TA’s catechol hydroxyl groups (Figure S18, SI). Electrochemical analyses (Figures S19 and S20, Tables S13 and S14, SI) reveal the system’s operational robustness: <5% power output fluctuation under ligand interference, achieved through the hole-capturing function of CNF photoanode, facilitating H^+^ reduction and strengthening separation of photogenerated charges. The ALCE-TGCNM configuration thus demonstrates remarkable resilience, maintaining dual-functional remediation performance (U(VI) removal > 89%, BPA degradation > 92%) while preserving stable electrochemical kinetics in ligand-rich environments. The catalyst has the maximal removal on both oxidation and reduction of BPA and uranium species with the average AOP treatment. Furthermore, the confirmation of the uranium oxidation state and the extent of reduction were carried out using bulk-sensitive XANES/EXAFS analysis.

We treated 100 mL of a solution containing 5 mg L^−1^ UO_2_^2+^ and 10 mg L^−1^ BPA under ambient sun light (20.1° N, 110.33° E) from 10:00 to 16:00 on 1 June 2025, in order to assess the ALCE-TGCNM system’s practical applicability (Figure S21). As depicted in Figure S22, Concurrent remediation capabilities were demonstrated by the ALCE system, with an efficiency of 96.6% in degrading BPA and 95.6% in eliminating uranium. Most notably, at 13:00, with the highest output, synchronous electricity generation was accomplished during the decontamination process, with a reported voltage of 2.26 V and current density of 1.83 mA cm^−2^ (Figure S23). The results suggest that the ALCE-TGCNM configuration works well under natural sun irradiation and can treat uranium–organic wastewater and convert solar energy at the same time, which is a double-edged sword. Removal efficiency was compared with previously reported materials in terms of uranium extraction [43] and BPA [44] removal performance (Table S15), and similarly various pseudo-first-order kinetic parameters, removal efficiency, and electrical outputs were compared across the four tested systems (Table 1). All values are reported as mean ± standard deviation (SD) from *n* = 3 independent triplicate experiments performed under identical conditions (10 mg L^−1^ U(VI), 20 mg L^−1^ BPA, 0.1 M Na_2_SO_4_, pH 5.0, simulated solar irradiation, AM 1.5 G, 100 mW cm^−2^). U(VI) removal reached 96.7 ± 1.3% and BPA degradation reached 97.7 ± 0.8% (mean ± SD, *n* = 3 independent triplicate experiments).

## 3. Materials and Methods

Text S5 (SI) contains full experimental details, including itemised listings of all reagents and consumables utilised throughout this research. All experiments were performed in triplicate, and results are reported as mean ± standard deviation. One-way ANOVA was used to assess the statistical significance of performance differences between experimental conditions (*p* < 0.05 considered significant)

### 3.1. Preparation of TGCNM Cathode

Details of the preparation of Ti_3_C_2_ MXene and TGCNM are given in Texts S6 and S7 (SI). Prior to the synthesis of the TGCNM cathode, the CF (carbon fibre) was cut into 2 × 5 cm pieces and subjected to continuous ultrasonic treatment using deionised water and ethanol [45]. Following the synthesis of the precursor, 40 mg of TGCNM and 5 mg of conductive carbon (acetylene black) were mechanically ground for 15 min to homogenise. After that, the substance was mixed with a polymer adhesive system that included half a millilitre each of ethanol and polyvinyl alcohol (PVA). Next, the TGCNM and acetylene black were ground evenly for 10 min to create a colloidal mixture. The surface of a 4 × 2 cm^2^ carbon fibre (CF) was uniformly coated with the produced suspension, making sure that the material was evenly dispersed. The TGCNM electrode was then made (morphological characterisation in the following steps) after being thermally cured at 60 °C for 6 h (Figure S29, SI). Parallel synthesis protocols generated control samples: Ti_3_C_2_, Ti_3_C_2_@GCN, and TGCNM. The synthesis protocol for the carbon/chitin nano film (CNF) is systematically outlined in Text S8 (SI). Subsequent structural and chemical analyses, including those of TGCNM heterojunctions and their carbon fibre composites, are fully described.

### 3.2. Photoanode Fabrication

The carbon-chitin nanofilm (CNF) photoanode component was prepared by casting a chitin/chitosan-based precursor film incorporating a conductive carbon component, followed by controlled thermal treatment to yield a conductive nanofilm, which was subsequently laminated onto the silicon photovoltaic cell to complete the tandem CNF/Si-PVC photoanode. Full experimental detail provided in the Supporting Information Text S7.

### 3.3. Establishment and Evaluation of ALCE

A composite photoanode and counter electrode system form the device’s central structure. The 2 × 4 cm^2^ Si PVC matrix and CNF film were combined to create the photoanode, while the TGCNM material system, heat-treated at 600 °C, made up the cathode. During the assembly process, the photoanode’s positive electrode is bonded to the CNF film using interface bonding technology, the negative electrode is connected to the cathode material using a conductive copper wire, and the photoanode is finally encapsulated using silicone encapsulation technology [46]. Photocatalytic evaluations employed quartz photoreactors (35 mL; Lianyungang Aoxin Quartz Technology Co., Ltd. Lianyungang, China) containing 0.1 m Na_2_SO_4_ electrolyte under AM 1.5 illumination (100 mW·cm^−2^), with pH modulation achieved via 0.1 m acid/base titrants. The uranium speciation was measured using an ICP-OES system (ThermoICPOES7200, ThermoFisher, Waltham, MA, USA, USAVarian720), and the removal of organic contaminants was monitored using a reverse-phase HPLC system (Agilent 1290 Infinity II LC–6460 Triple Quadrupole MS: Agilent Technologies, Inc., Santa Clara, CA, USA). Potentiostat readings were integrated with in-situ electrochemical monitoring (CH1730E, CH Instruments, Inc., Austin, TX, USA) and multi-matrix validation. The experimental conditions were controlled in triplicate, as reflected by all measures.

## 4. Conclusions

The ALCE system described in this study performs U(VI) reduction, organic pollutant degradation, and electricity generation concurrently. CNF generates e^−^/h^+^ pairs upon the absorption of photons with wavelengths below 412 nm, while the Si PVC harvests longer wavelengths to establish a self-bias that drives electrons to the TGCNM cathode for continuous current. H^+^ and •OH act synergistically to oxidise BPA and dissociate UO_2_^2+^, BPA complexes, while electrons reduce U(VI) to insoluble U(IV) at the cathode. Under simulated sunlight, ALCE achieved simultaneous removal efficiencies of 96.7% for UO_2_^2+^ and 97.7% for BPA alongside a sustained power output of 1.82 mW·cm^−2^. Future work should focus on further optimisation of the TGCNM cathode composition and heterojunction interface, scale-up to larger or modular device configurations, development of continuous-flow reactor architectures suitable for practical deployment, and validation using authentic radioactive/industrial wastewater matrices with extended durability testing. Relative to previously reported self-driven photocatalytic/photoelectrocatalytic uranium recovery systems, the present TGCNM-based system achieves comparable-to-superior U(VI)/BPA removal while combining recovery, organic degradation, and electricity generation without reliance on noble-metal co-catalysts. The remaining limitations include a power density at the lower end of the comparator range and the absence of pilot-scale or real-wastewater validation.

## Figures and Tables

**Table 1:** Comparison of pseudo-first-order kinetic parameters, removal efficiency, and electrical output across the four tested systems: dark adsorption, simple photolysis, ALCE-CF, and ALCE-TGCNM.

System	k (U(VI)) (min^−1^)	R^2^ (U(VI))	k (BPA) (min^−1^)	R^2^ (BPA)	U(VI) Removal (%)	BPA Removal (%)	Power Density (mW cm^−2^)	*n*
**Adsorption**	0.008	0.941	0.003	0.918	24.1 ± 2.1	8.3 ± 1.4	-	3
**Photolysis**	0.015	0.963	0.011	0.947	38.6 ± 2.8	29.7 ± 2.3	-	3
**ALCE-CF**	0.034	0.978	0.028	0.965	71.4 ± 2.0	68.2 ± 1.9	0.34 ± 0.03	3
**ALCE-TGCNM**	0.061	0.992	0.052	0.988	96.7 ± 1.3	97.7 ± 0.8	0.91 ± 0.05	3

## Data Availability

Data will be made available on request.

## Supplementary Materials

The following supporting information can be downloaded at: https://www.mdpi.com/article/10.3390/ijms27198680/s1.
