# Supplementary material for: An Integrated Self-Powered Photoelectrocatalytic System for Quadruple-Function Water Remediation: Organic Degradation, Radionuclide Recycling, Energy Recovery, and Toxicity Evaluation
Source: Int J Mol Sci. 2026 Sep 28;27(19):8680. doi: 10.3390/ijms27198680 (PMC13665024; doi:10.3390/ijms27198680)
Supplement: Supplementary file 1 [file ijms-27-08680-s001.zip › ijms-4580447-supplementary.pdf]

Supporting Information

Text S1. Characterization

The structural and physicochemical properties of the synthesized materials were systematically characterized through advanced analytical techniques. Morphological features and elemental distribution profiles were captured through field-emission scanning electron microscopy (FE-SEM, Hitachi SU8010 HORIBA-EMX) coupled with energy-dispersive X-ray spectroscopy. FE-TEM Model: A300-10/12: BC250524-61: Instrument model: FEI Talos F200X with energy spectrum of super X acceleration voltage of 200KV. Crystalline phase identification was performed via X-ray diffraction (Dmax2500) using Cu Kα radiation (λ = 0.15406 nm) across a 2θ range of 10-80° with a scanning rate of 5°/min. Complementary surface chemical analysis employed X-ray photoelectron spectroscopy (Thermo ESCALAB 250XI) under ultrahigh vacuum conditions (5×10^{-10} mbar) with monochromated Al Kα excitation (1486.6 eV), where binding energies were calibrated against adventitious carbon at 284.8 eV. Porosity characteristics were quantified through nitrogen adsorption-desorption isotherms at 77 K (ASAP 2020 analyzer), with specific surface areas calculated via the Brunauer-Emmett-Teller model and pore size distributions determined through the Barrett-Joyner-Halenda method following 12-hour degassing at 150°C. Electrochemical evaluations were conducted using a 50 ml 0.1 M Na_2SO_4 solution as the electrolyte. Cyclic voltammetry (CV) and electrochemical impedance spectroscopy (EIS) tests were conducted using a three-electrode system, with the working electrode being TGCNM/CF, the counter electrode was a Pt foil, and the reference electrode was an Ag/AgCl electrode. The initial potential for EIS was -0.4 V, with a frequency range of 0.01 to 10^5 Hz. The potential range for CV testing was -1.5 V to 0.5 V. Photo-response measurements employed a solar-simulated light source (300 W Xe lamp, AM 1.5G filter) calibrated to 100 mW·cm^{-2} irradiance using a silicon reference cell. The optical properties of catalysts and pollutants were measured using UV-Vis spectrophotometer under Xenon lamp. The HPLC measurement for the analysis of bisphenol A(BPA) pollutant under the experimental conditions of mobile phase (1:1) for the methanol: acetophenone for the separation and purification of medium under the scan rate of 20 μL on C-18 filter column. The optical recombination properties of the electrocatalyst have been calculated using photoluminescence (PL) spectra embedded Fluorescence spectroscopy. The maximum wavelength of as-prepared catalyst which shows higher removal showcase higher pretending of lowered half-life period which tends to have a greater number of active sites present in it. Higher the void width can be measured using the BET isotherm on an average basics of the higher-level removal of BPA/U(VI) manifest to have the lower recombination and enhanced electron separation. The analytical method here used for measuring intrinsic properties of solid photocatalysts using the luminescence studies, UV-vis adsorption and HPLC-MS, respectively. The reduction of uranium with various oxidation states were analyzed using ICP-OES .

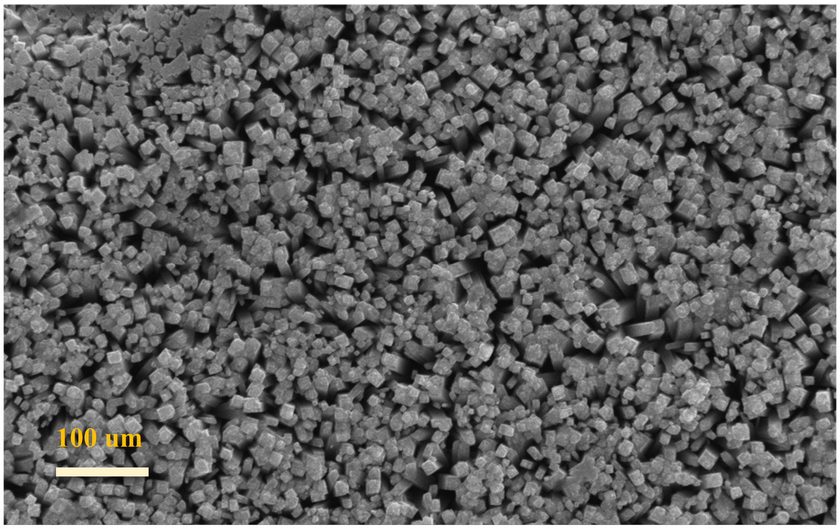

Figure S1. SEM image of CNF.

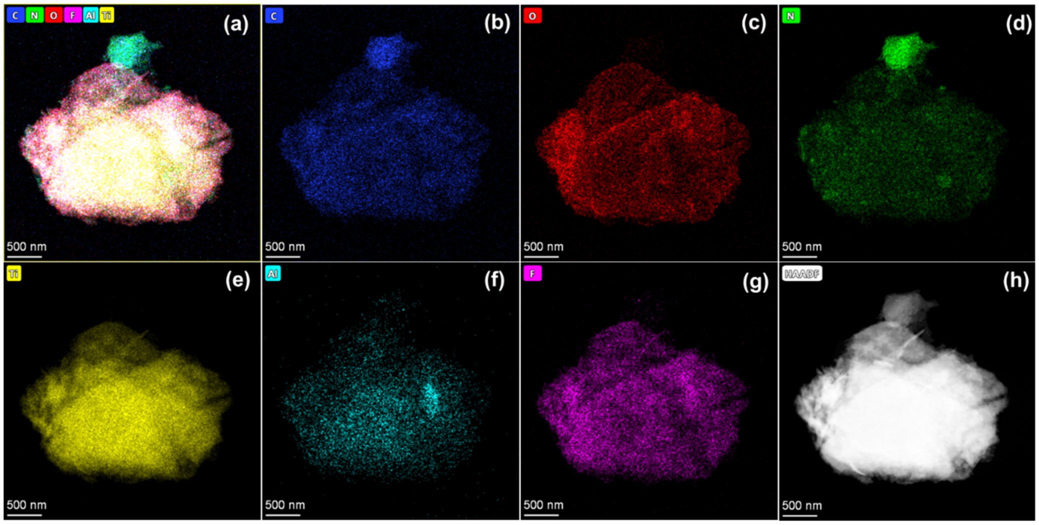

Figure S2. Surface morphology (**a**–**e**), SAED pattern (**f**), diffraction lines (**g**,**h**) and the mapping images of different elemental distribution of as-prepared TGCNM photocatalyst.

Text S2. ALCE performance using various cathodes

Under dark conditions, TGCNM exhibited optimal adsorption performance, achieving 50.7% UO_2^{2+} removal and 51.5% BPA degradation (**Figures S14a-b**), potentially attributable to UO_2^{2+} complexation with surface carboxyl groups on MXene. Under AM 1.5 illumination, the material demonstrated significantly enhanced efficiency, removing 96.8% of UO_2^{2+} within 120 minutes (rate constant k ≈ 0.028 min^{-1}), representing a 7.7-fold improvement over CF (**Figures S14c-d**). The synergistic combination of TGCNM exceptional adsorption capacity and ALCE's electron-driving properties facilitated efficient electron transfer to cathodes for UO_2^{2+} reduction. For BPA degradation, 97.8% removal was achieved under identical illumination with k ≈ 0.087 min^{-1}, corresponding to 6.6-fold enhancement versus CF (**Figures S14e-f**). This performance enhancement originated from internal bias generated through transmitted light capture by Si PVC, which improved electron-hole pair separation efficiency in CNF. Consequently, more photogenerated holes (h^+) participated in oxidizing surface-adsorbed H_2O/organics rather than recombining with electrons. The self-powered ALCE exhibited notable photovoltaic parameters: J_{SC} = 1.09-1.43 mA·cm^{-2}, V_{OC} = 2.03-2.47 V, P_{max} = 1.08-1.82 mW·cm^{-2}, and FF = 46.2-53.0% (**Figures S14g-h**, **Table S2**). The removal rate (η) and removal rate (k) as well as P_{max} are calculated as follows:

$$\eta = C/C_0 * 100\%$$

$$k = \ln(C/C_0)$$

$$P_{max} = FF * J_{SC} * V_{OC}$$

where C is the initial substrate concentration, and C_0 is the substrate concentration at time t.

Text S3. Experiments on the removal of pollutants using various systems

Photolysis uranium elimination describes the spontaneous decomposition of uranyl species in solution without electrode activation. CNF-ads quantifies the adsorptive uranium uptake capacity of Carbon/chitosan nanofilm photoanodes under dark conditions. CNF-Photocatalysis uranium elimination characterizes the light-driven uranyl remediation capability of CNF photoanodes under optical excitation. Dark-CF measures the uranium adsorption performance of carbon felt cathodes in non-illuminated environments. Dark-TGCNM evaluates the uranyl ion sequestration capacity of MXene-modified cathodes under dark operation.

ALCE-CF designates the autonomous photo electrocatalytic uranium extraction efficiency in self-powered systems combining CNF photoanodes with pristine CF cathodes under illumination. ALCE- TGCNM specifies the enhanced uranyl removal performance achieved through integration of CNF photoanodes with TGCNM cathodes in equivalent self-powered photo- electrocatalytic configurations. The excess of various concentration which causes the higher removal capacity towards the photo anode and cathode with the higher limitation to that of electrolyte solution. The major species involved in the reduction of uranium which have the capacity to oxidize BPA as well as the maximal removal technique under the optimal conditions. The CF which causes the transfer of electrons to the photocathode for the movement of e^{-s} in the surface of the electrode which easily the uranium got reduced to the in-soluble species of U(IV) with the higher proportion compared to the soluble uranium U(VI) species. Herein, the formation of species which clearly shows that the ALCE system with the higher capacity to reduce the reduction part of both BPA and uranium species. The maximum removal of BPA on optimal conditions shows that the higher percentage of removal efficiency on it.

Deconvolution of the O 1s spectrum (**Figure S3a**) resolved two distinct contributions at 528.7 eV (Ti-O-Ti lattice oxygen of Ti_3C_2) and 530.7 eV (oxygen-deficient sites in CS). Notably, the Ti 2p profile (**Figure S3b**) displayed symmetrical doublets with binding energies of 457.4 eV (Ti $2p_{3/2}$) and 463.2 eV (Ti $2p_{1/2}$), aligning with the Ti^{3+} state in crystalline Ti_3C_2. These spectroscopic signatures collectively confirm the effective hydrothermal fabrication of CNF. The maximum peak length of the higher ratio resembles the breakage of bond length towards the higher removal capacity due to chemisorption between the adsorbent and adsorbate with the exposure of light and intensity with

Photoelectrochemical testing under 0.5 V vs. Ag/AgCl bias yielded an anodic current density of ~0.9 $mA{\cdot}cm^{-2}$ for the TNR photoanode (**Figure S12a**). Continuous operational stability assessments via chronoamperometry (**Figure S12b**) demonstrated retained photo-response efficiency (>92% retention) over prolonged illumination cycles, validating interfacial charge transfer robustness [35].

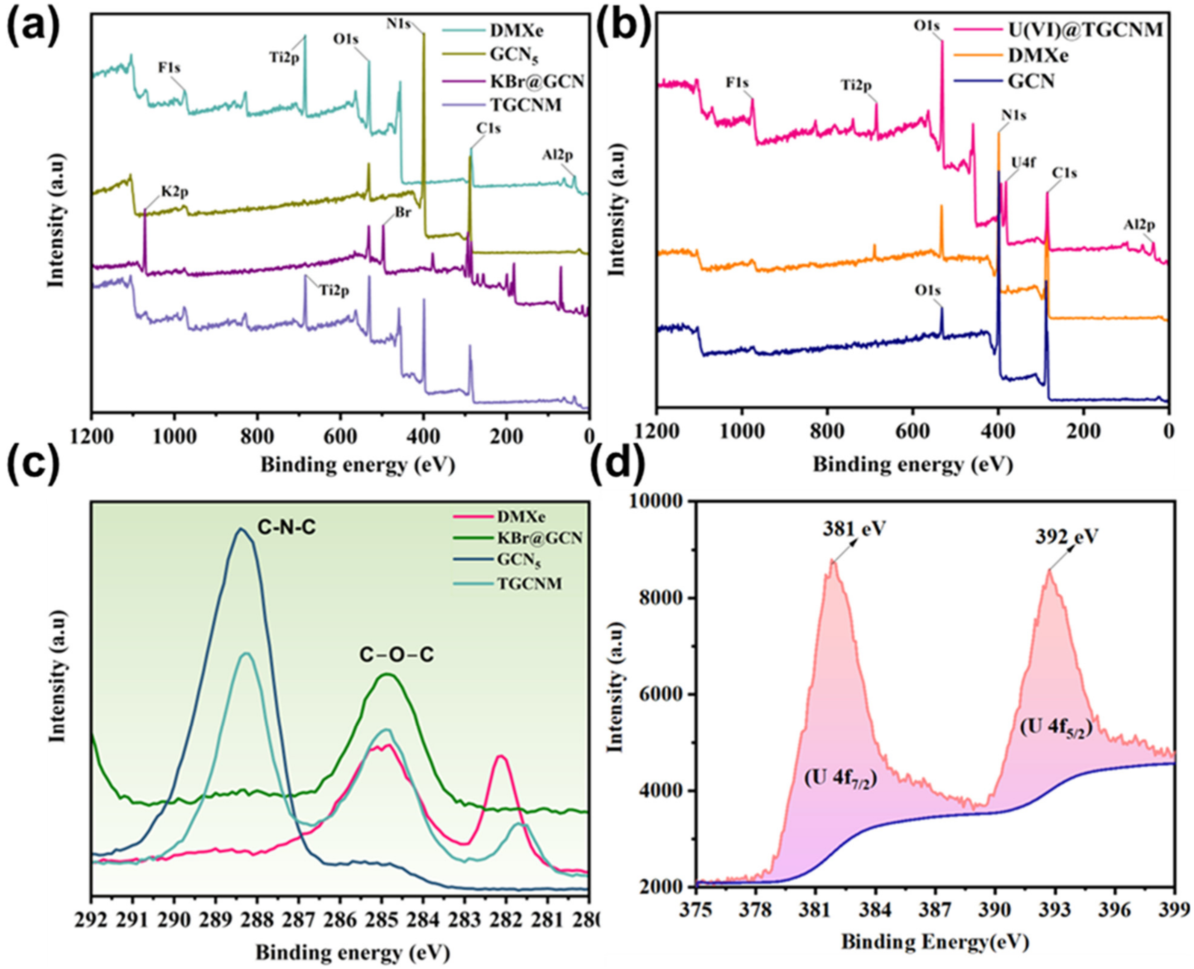

Figure S3. (**a**,**b**) Survey spectra of before and after reduction of UO_2^{2+} by photocathode TGCNM; (**c**,**d**) C 1s spectra after the adsorption of UO_2^{2+} under Dark and ALCE conditions.

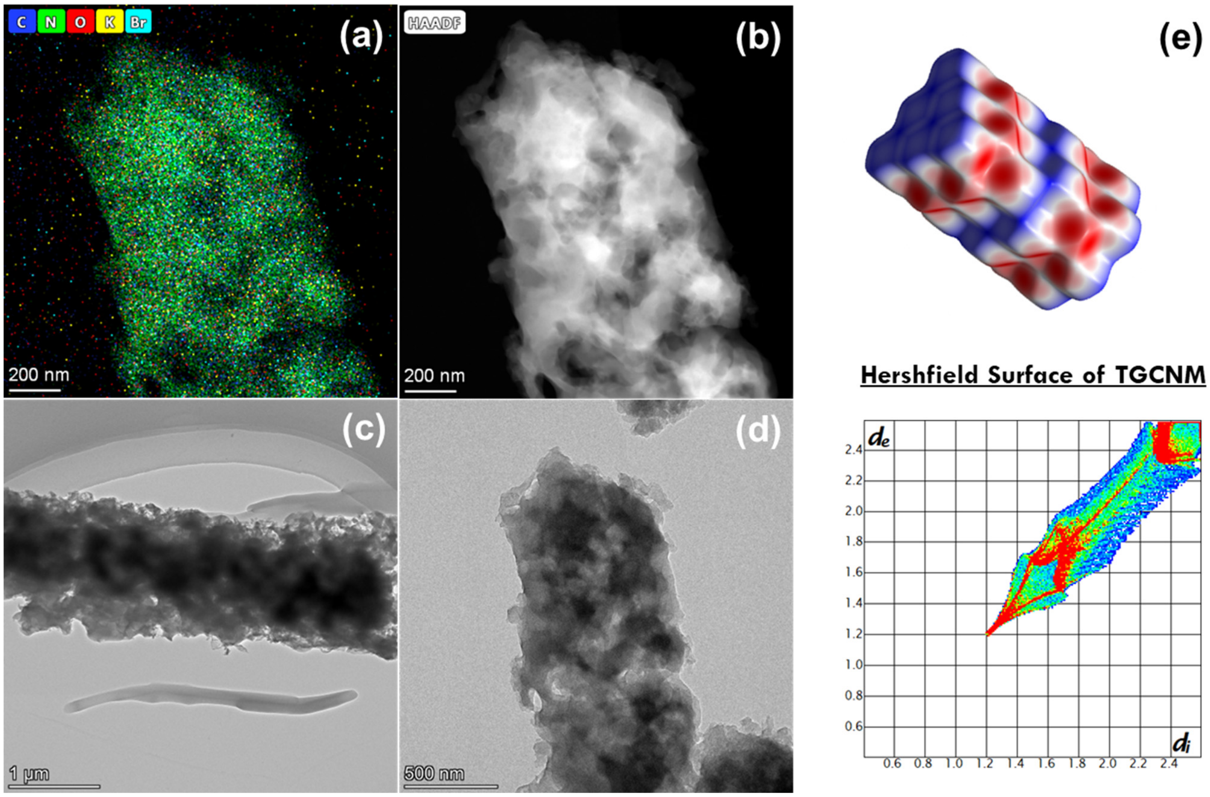

Figure S4. TEM image of photoanode (a) EDX and (b) HAADF TGCNM (c–d) TGCNM photoanode, and (e) 2D Hirshfeld surface Analysis.

Text S4. Degradation pathways and toxicity assessment

To further elucidate the potential degradation pathways of tetracycline hydrochloride (BPA), intermediate products generated during the degradation of this representative recalcitrant organic pollutant were systematically analyzed by liquid chromatography-mass spectrometry (HPLC-MS), as shown in **Figure S21**. During photodegradation, BPA underwent structural transformation and ultimate mineralization to inorganic small molecules via three primary pathways (**Figure S22**). In Pathway I, BPA experienced dehydration under the action of h^+ to form P2 (m/z = 429). Pathway II involved the hydroxyl radical (•OH)-mediated oxidation of BPA, yielding intermediate P7 (m/z = 475) [36]. In Pathway III, h^+ induced the demethylation of the BPA molecule, resulting in P1 (m/z = 417). These initial intermediates subsequently underwent further decomposition processes including ring cleavage, deoxygenation, dehydrogenation, and deamination which forms monoaromatic or branched-chain molecules. Ultimately, these intermediates were oxidized to inorganic small molecules (including CO_2 and H_2O), completing the mineralization process. The oxidation of BPA which has the higher formation of by-products with less toxic nature shows that the ALCE with the AM 1.5 illumination under the simulated sunlight has the higher capacity to hold the maximum removal rate of the BPA with the higher point of the zero-charge potential as shown in **Figure S21.** The removal rate of the BPA with the retention time rate of around 2.75 min with the higher peak area got oxidized.

Acute toxicity of BPA and its transformation intermediates was assessed using the T.E.S.T software tool. BPA exhibited an oral LD_{50} in rats of 1540.4 mg/kg (**Figure S23a**). Only certain intermediates (e.g., P9, P10) demonstrated higher toxicity than BPA itself; however, these are transient products and not final degradation species. The most toxic intermediates identified were predominantly primary and secondary metabolites. In contrast, the majority of downstream intermediates existed in a non-toxic threshold. Regarding developmental toxicity (**Figure S23b**), toxicity was confined to P2 and P15, with most intermediates falling within safe limits. Mutagenicity predictions (**Figure S23b**) indicated that while some degradation products retained toxicity, the overall toxicity of the parent BPA diminishes through degradation. Furthermore, downstream intermediates were largely devoid of developmental toxicity. The BPA-intermediates which itself has the higher removal rate with percentage of removal with it maximize part of the major by-product removal on the successful reduction which have existence of precipitation and hydrolysis of uranium at higher pH. The BPA itself with benzene ring got ruptured and bind to the functional moiety of the photocathode with the TGCNM material coated on the surface. The retention time of BPA which has the capacity to reliable on ALCE AM1.5 illumination at operational parameters.

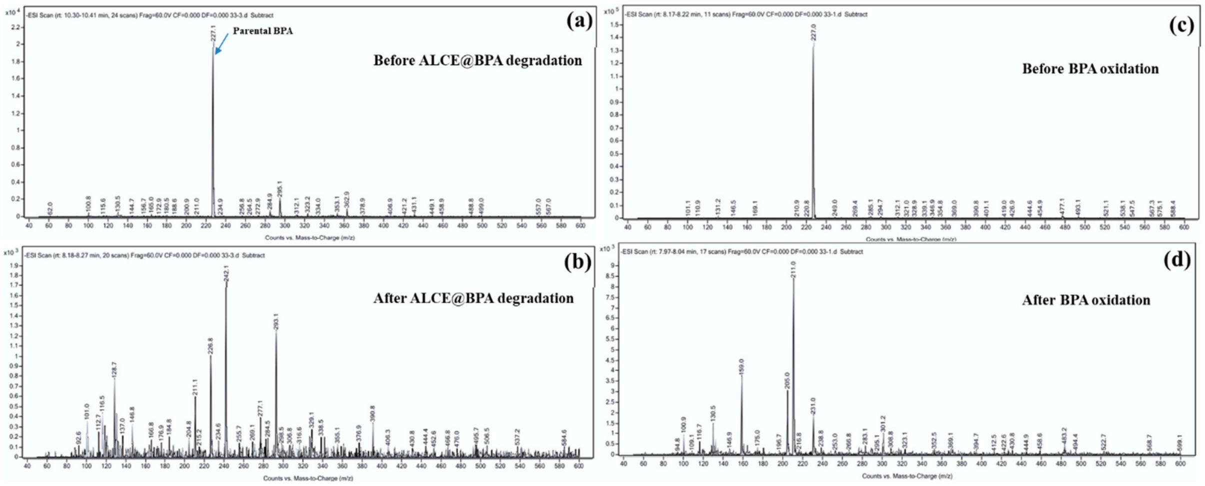

Figure S5. (a,b) HPLC-MS spectra of ALCE@BPA intermediates, (c,d) Before and after oxidation of BPA on ALCE 1.5 irradiation with the light intensity.

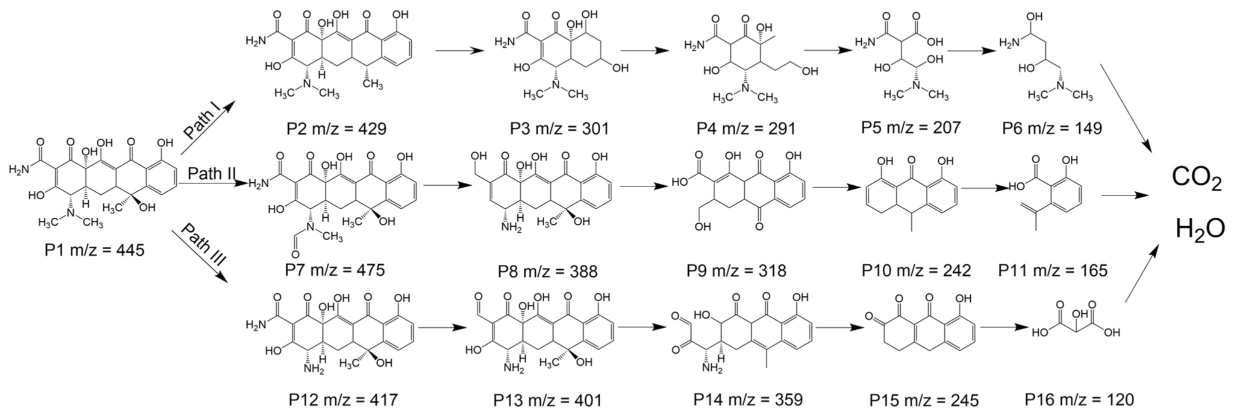

Figure S6. Proposed degradation pathways of BPA**.**

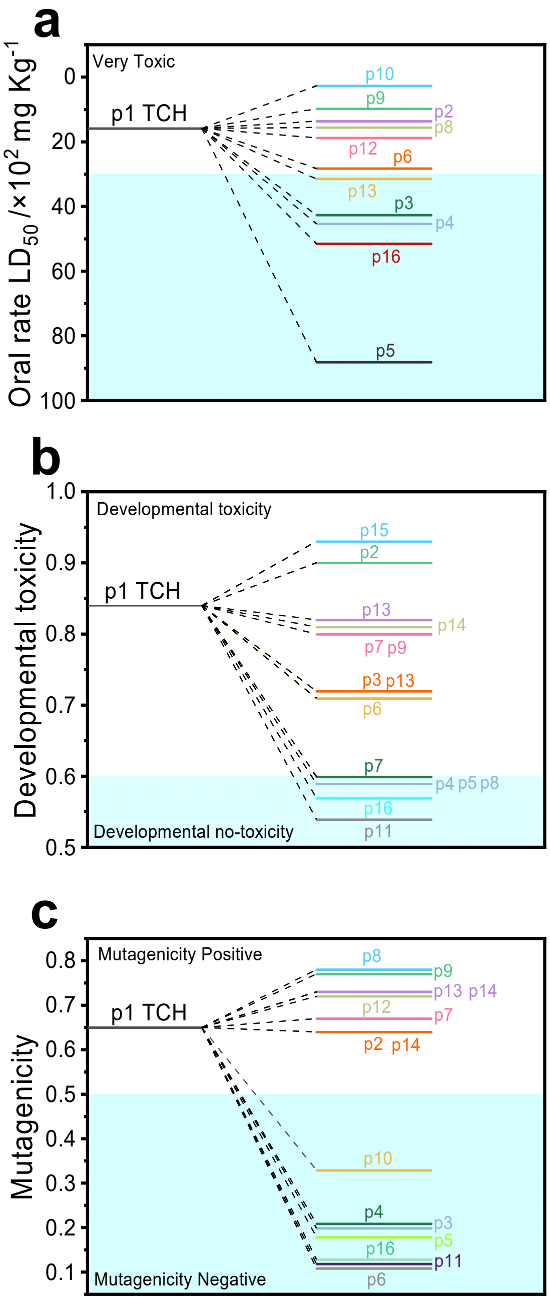

Figure S7. (a) Acute toxicity, (b) developmental toxicity, and (c) mutagenicity of the intermediates during TCH(BPA) degradation.

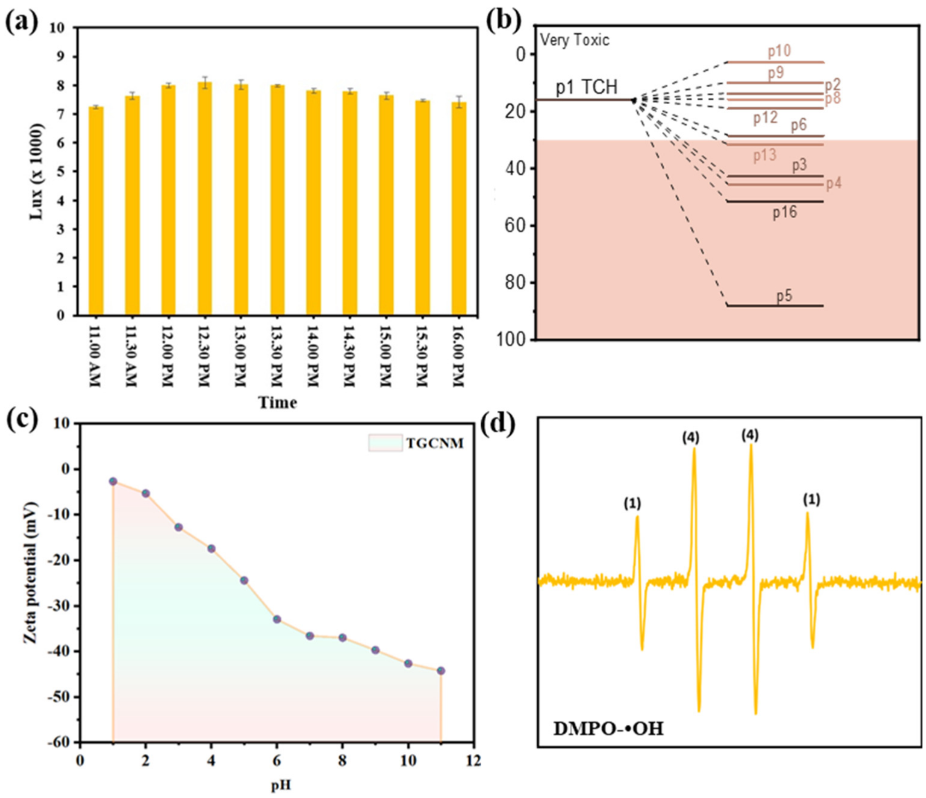

Figure. S8. (a) Sunlight intensity over experiment time under light irradiation, (b) Acute toxicity of the intermediates during TCH (BPA) degradation, (c) Zeta potential, and (d) ESR signals of DMPO-•OH by ALCE under AM 1.5 illumination.

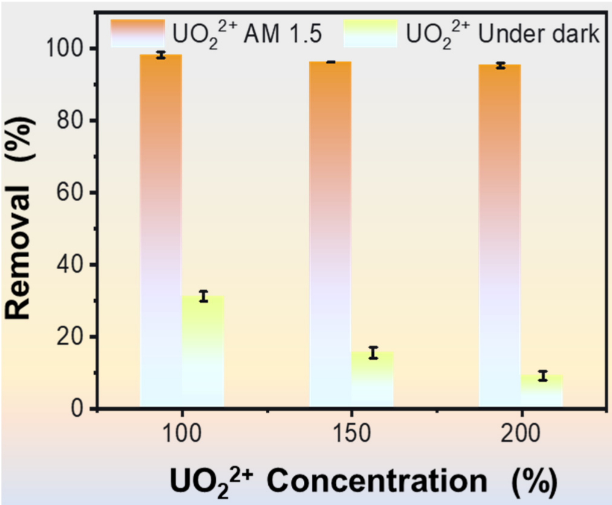

Figure S9. Effect of high concentration UO_2^{2+} on ALCE performance under light and dark condition.

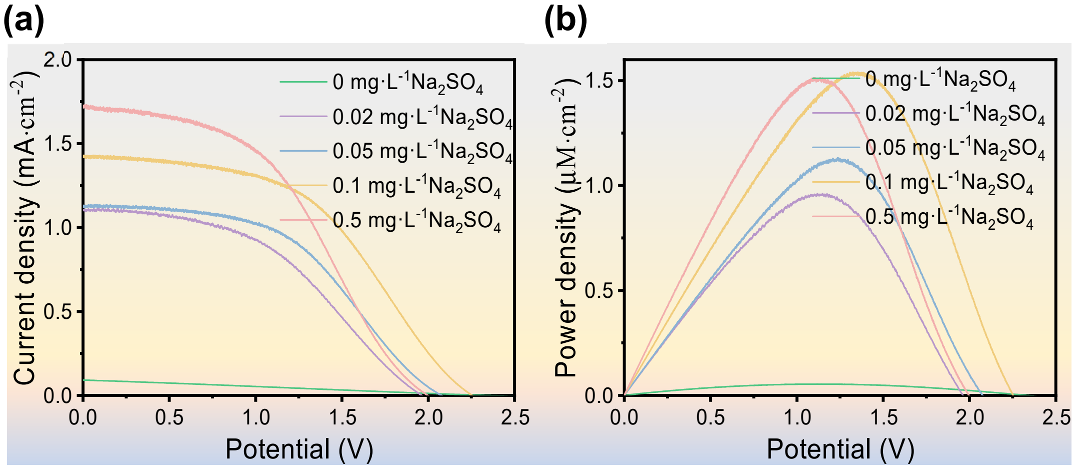

Figure S10. (a) *J-V* curves and (b) power output curves under AM 1.5 illumination at various electrolyte solution concentrations. Conditions: C_0 (UO_2^{2+}) = 10 mg·L^{-1}, C_0 (BPA) = 10 mg·L^{-1}, pH = 5, T = 298K, AM 1.5 illumination.

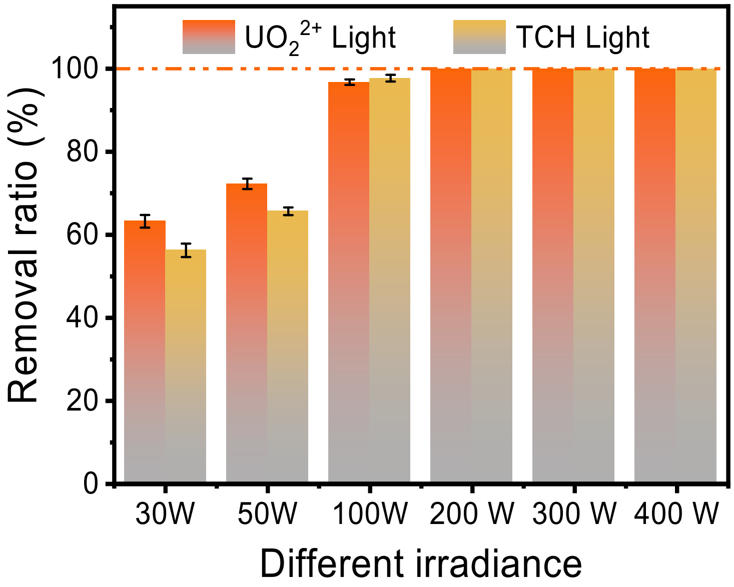

Figure S11. Removal performance of ALCE for UO_2^{2+} and BPA under different irradiance levels.

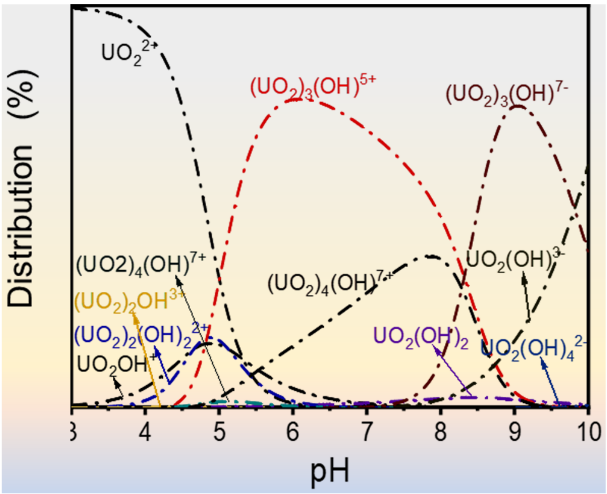

Figure S12. Simulated results of uranyl forms as a function of solution pH calculated using the software Visual MINTEQ. Conditions: C_0 (UO_2^{2+}) = 10 mg·L^{-1}, [Na_2SO_4] = 0.1 M.

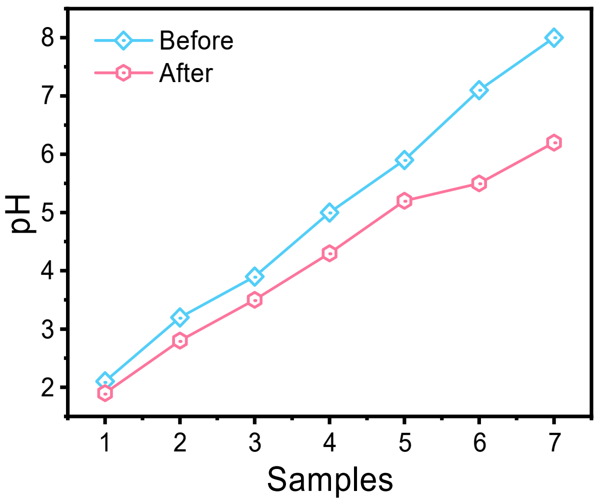

Figure S13. ALCE treats changes in solution pH before and after treatment at different initial pH levels.

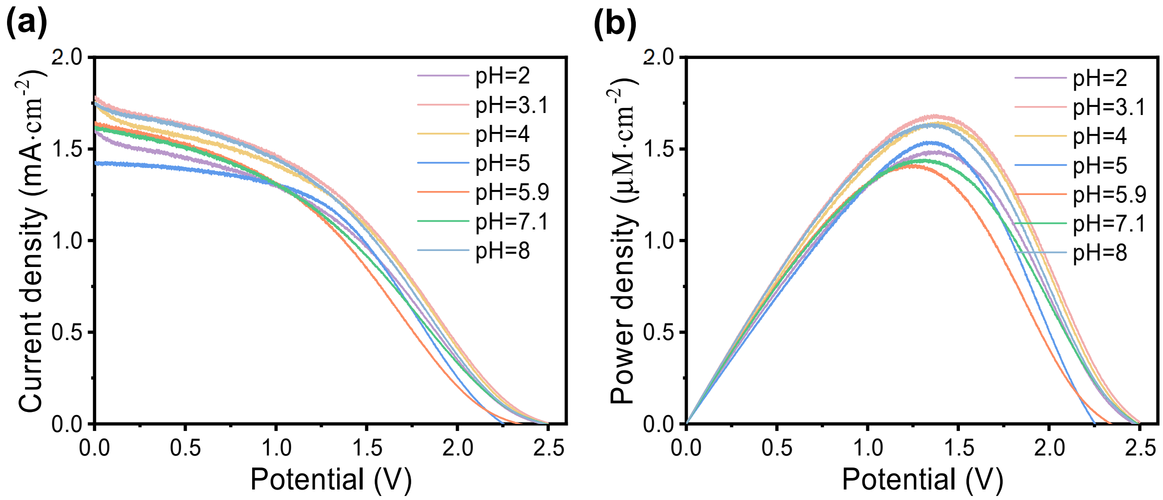

Figure S14. (a) J-V curves and (b) power output curves at various pH conditions: C_0 (UO_2^{2+}) = 10 mg·L^{-1}, C_0 (BPA) = 10 mg·L^{-1}, T = 298K, AM 1.5 illumination.

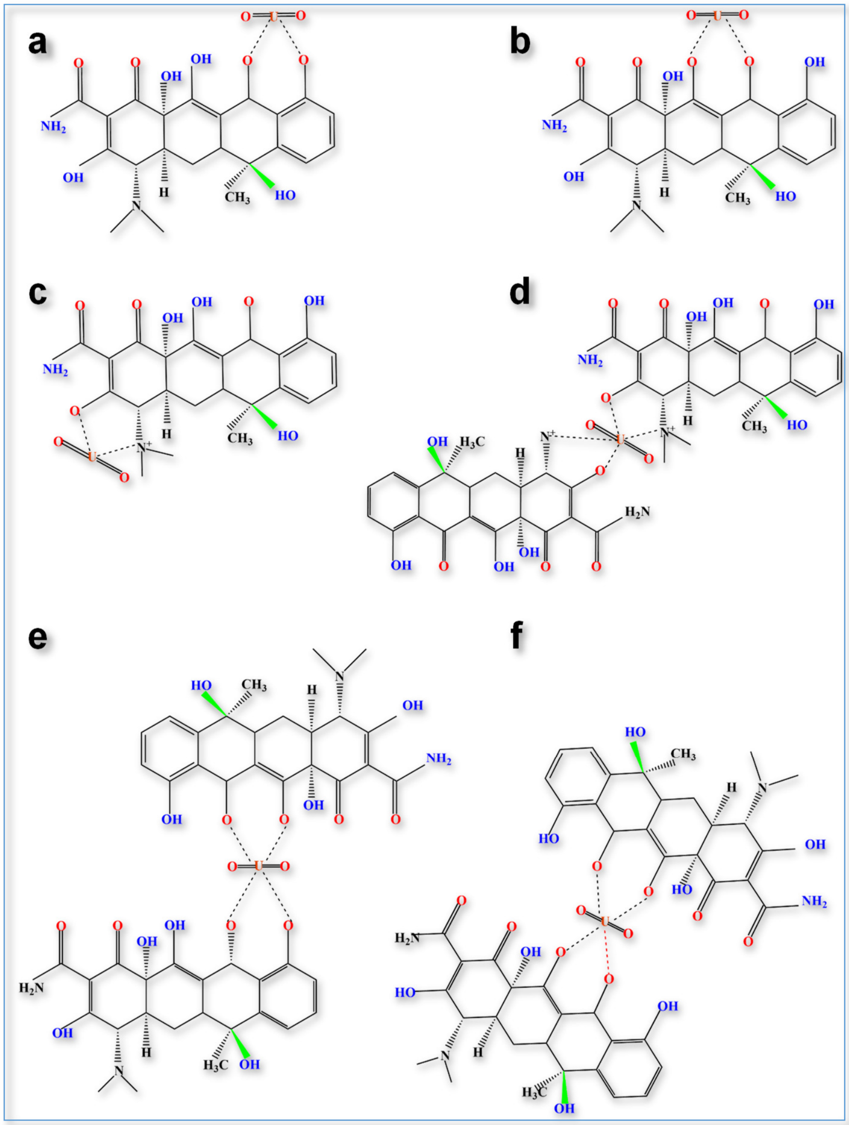

Figure S15. Possible coordination forms of UO_2^{2+} (a–c) and BPA (d–f) intermediates formed during the removal process.

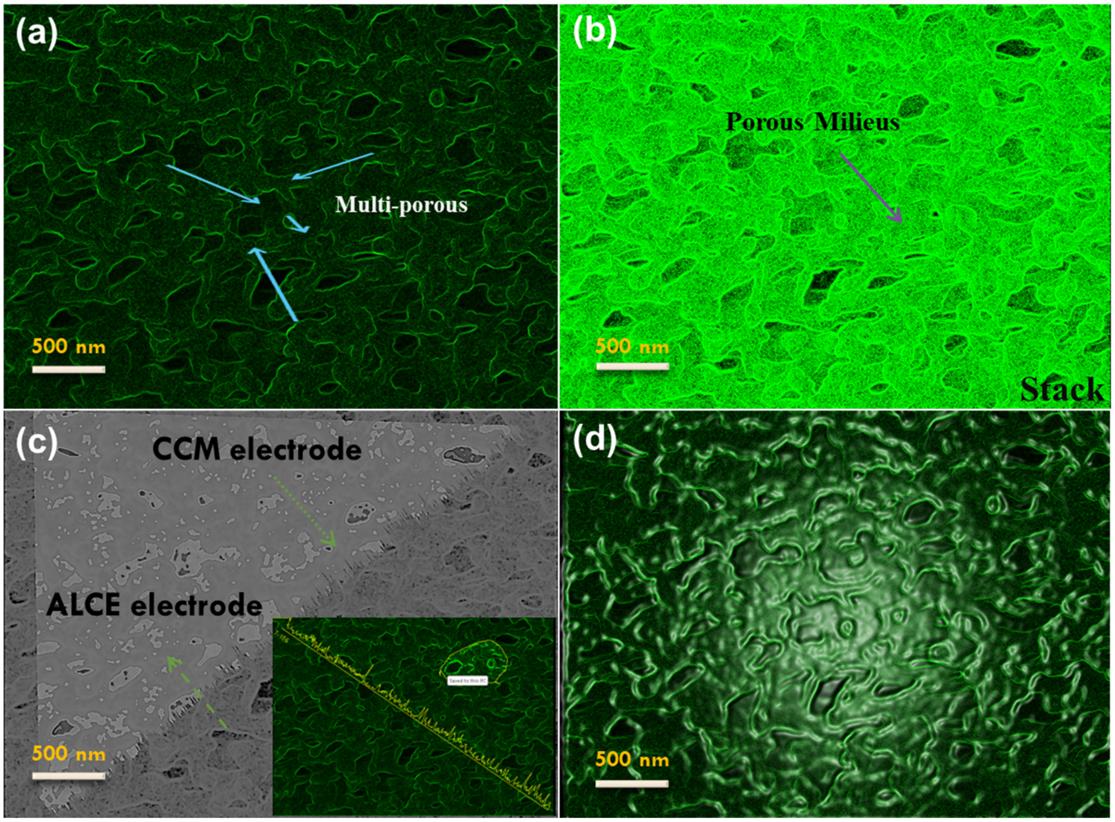

Figure S16. (a,b) Porous milieus, (c,d) Line profile electrode surface with various porous distribution on removal of UO_2^{2+} and BPA by ALCE system at different UO_2^{2+} concentrations. Conditions: C_0 (BPA) = 10 mg·L^{-1}, pH = 5, T = 298K.

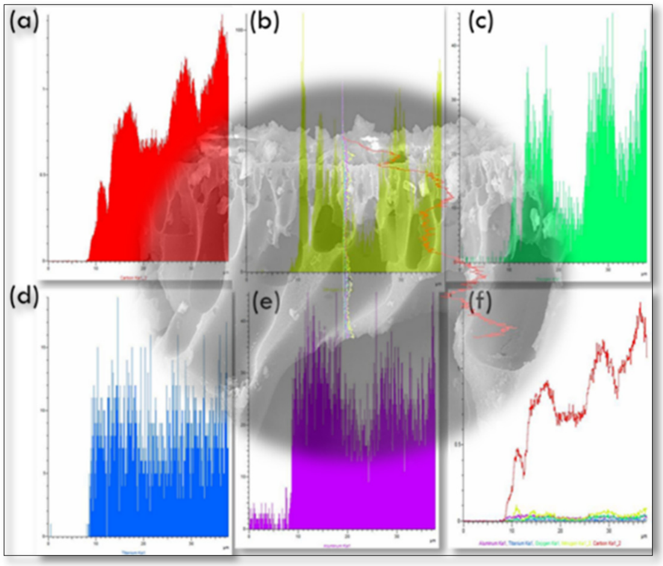

Figure S17. EDS-Mapping spectrum of the TGCNM after uranium adsorption under SEM model ((a) Carbon; (b) Nitrogen; (c) Oxygen; (d) Titanium; (e) Aluminum, and (f) EDS spectra, respectively).

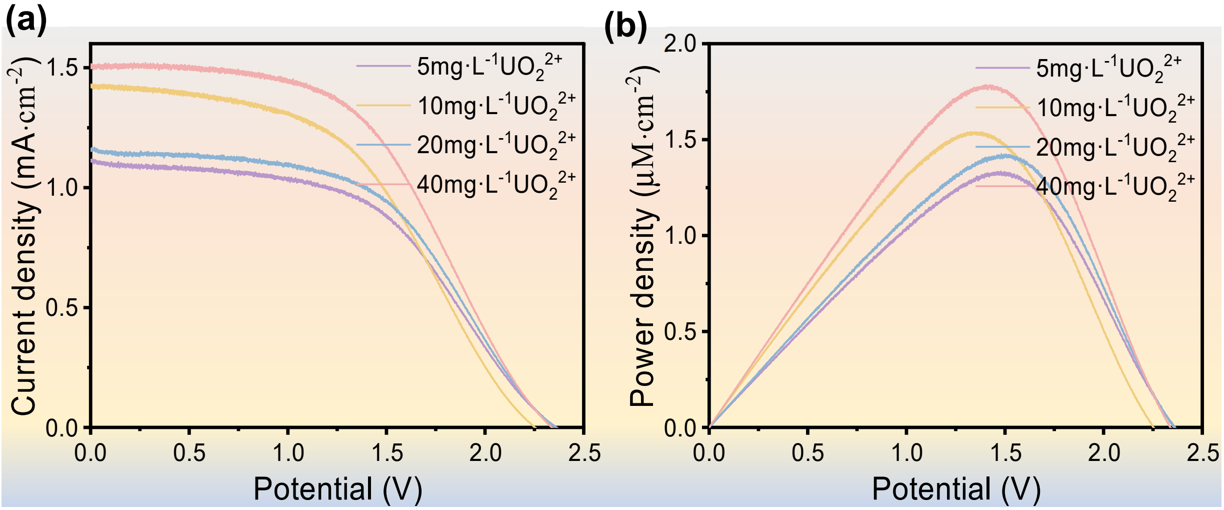

Figure S18. (a) *J-V* curve and (b) power output curve at various BPA concentrations. Conditions: C_0 (UO_2^{2+}) = 10 mg·L^{-1}, pH = 5, T = 298K, AM 1.5 illumination under ALCE system.

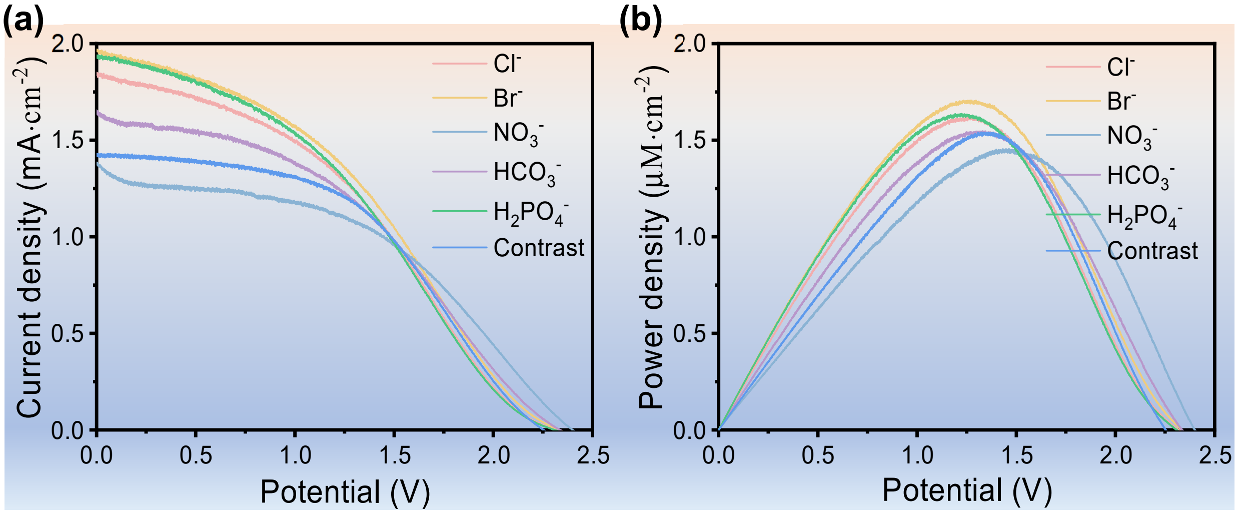

Figure S19. In the coexistence of various anions, (a) *J-V* curves and (b) power output curves. Conditions: C_0 (UO_2^{2+}) = 10 mg·L^{-1}, C_0 (BPA) = 10 mg·L^{-1}, pH = 5, T = 298K, AM 1.5 illumination.

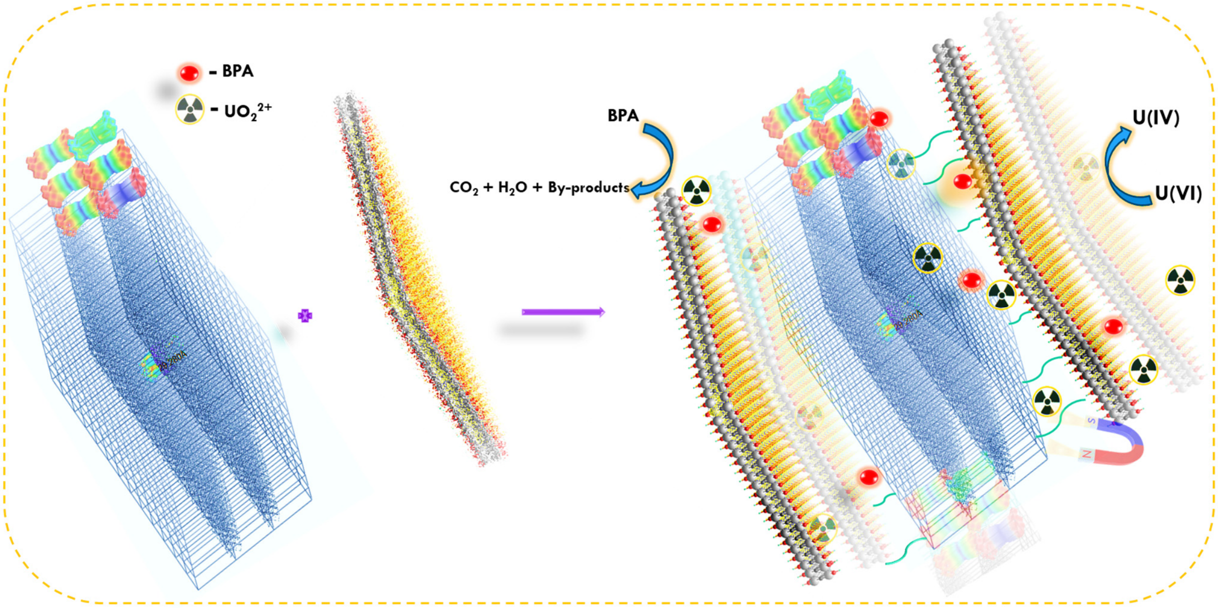

Figure S20. Possible mechanistic approach on dual-driven ALCE milieus on removal of BPA and UO_2^{2+} under AM 1.5 illumination.

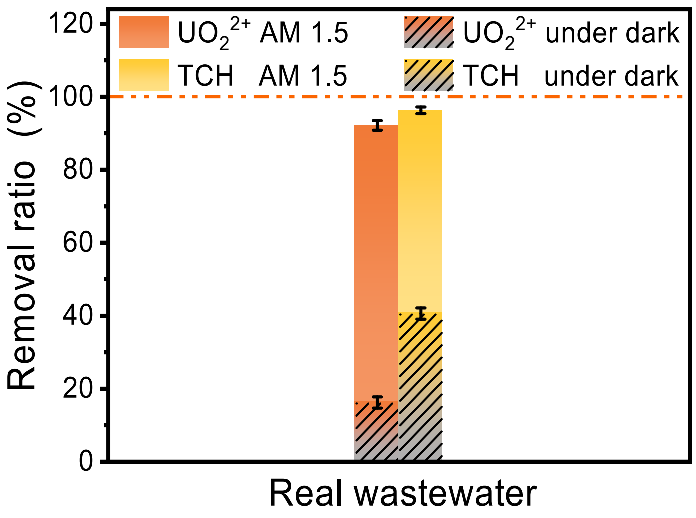

Figure S21. Seawater from Qiongzhou Strait, Hainan (South China Sea) (pH = 8.1, UO_2^{2+} = 5 mg·L^{-1}, BPA = 10 mg·L^{-1}).

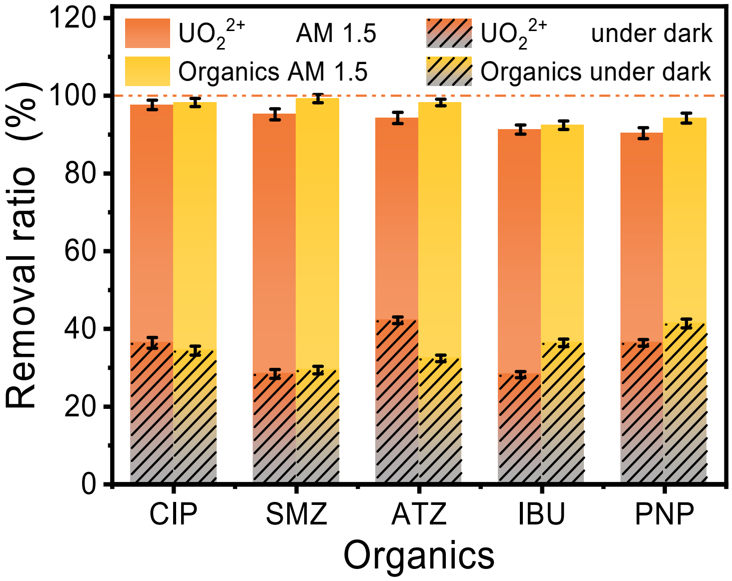

Figure S22. Removal ratios of UO_2^{2+} and organic compounds in the treatment of various organic-UO_2^{2+} mixtures.

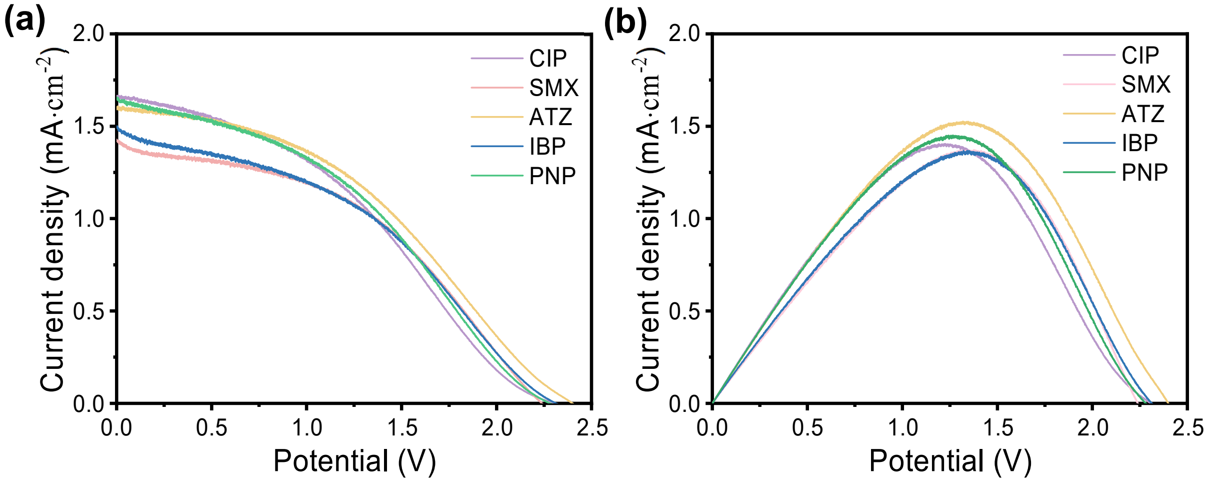

Figure S23. In the coexistence of various organics, (a) *J-V* curves and (b) power output curves. Conditions: C_0 (UO_2^{2+}) = 10 mg·L^{-1}, C_0 (organic) = 10 mg·L^{-1}, pH = 5, T = 298K, AM 1.5 illumination.

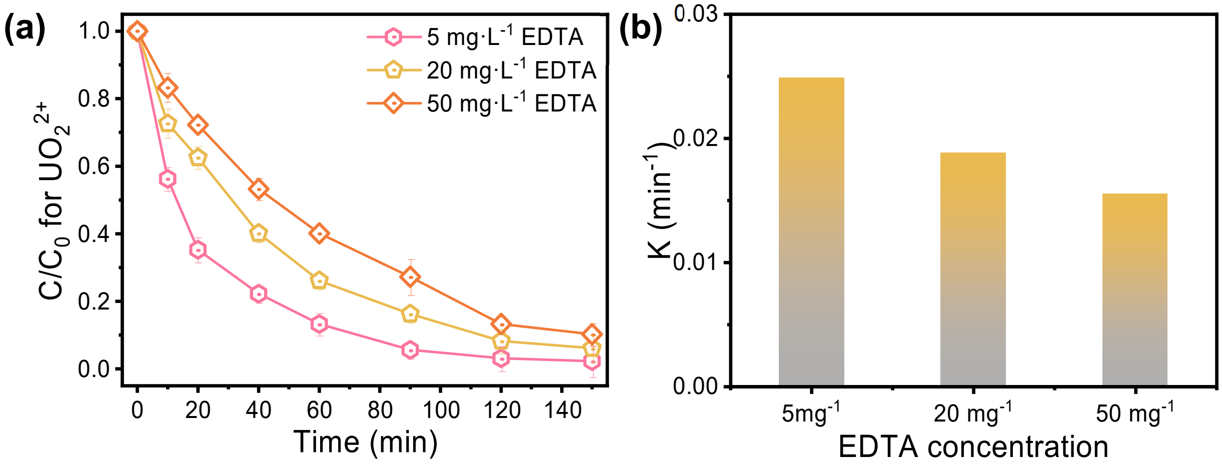

Figure S24. (a) Removal performance of UO_2^{2+} and (b) the rate constant k by ALCE-TGCNM system at different EDTA concentrations.

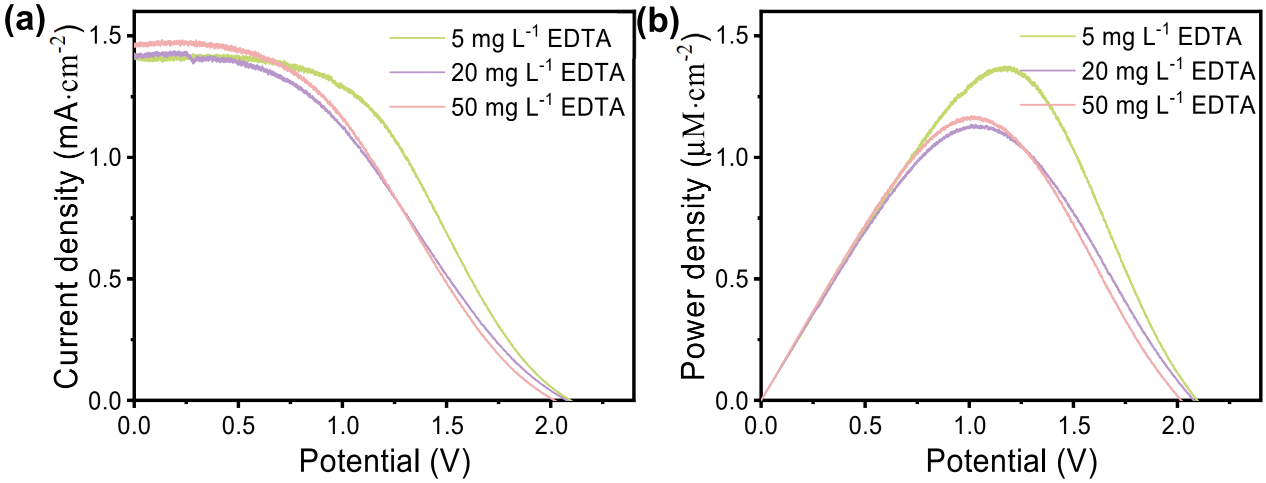

Figure S25. (a) J-V curves and (b) power output curves at various EDTA concentrations. Conditions: C_0 (UO_2^{2+}) = 10 mg·L^{-1}, pH = 5, T = 298K, AM 1.5 illumination.

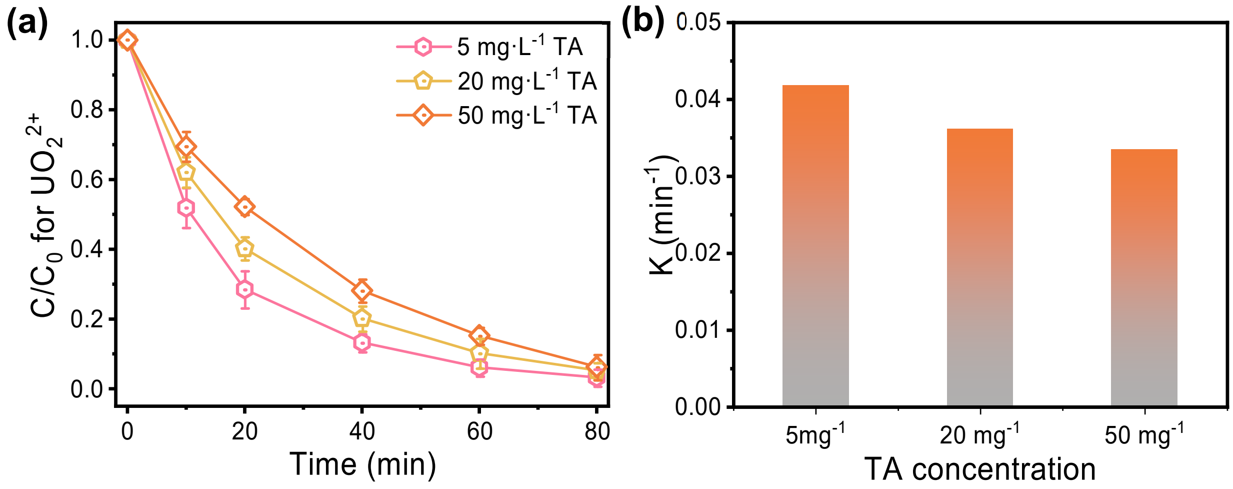

Figure S26. (a) Removal performance of UO_2^{2+} and (b) the rate constant k by ALCE-TGCNM system at different TA concentrations.

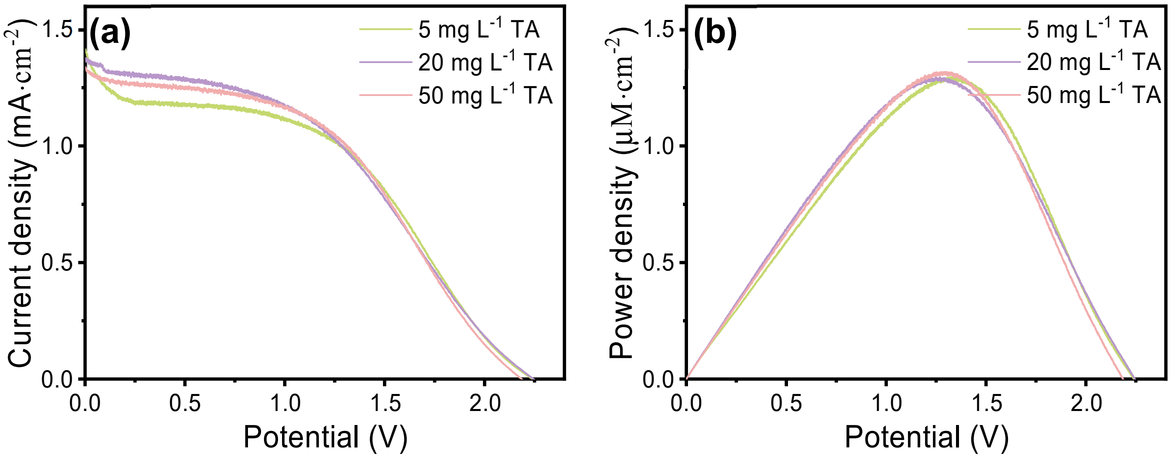

Figure S27. (a) J-V curves and (b) power output curves at various EDTA concentrations. Conditions: C_0 (UO_2^{2+}) = 10 mg·L^{-1}, pH = 5, T = 298K, AM 1.5 illumination.

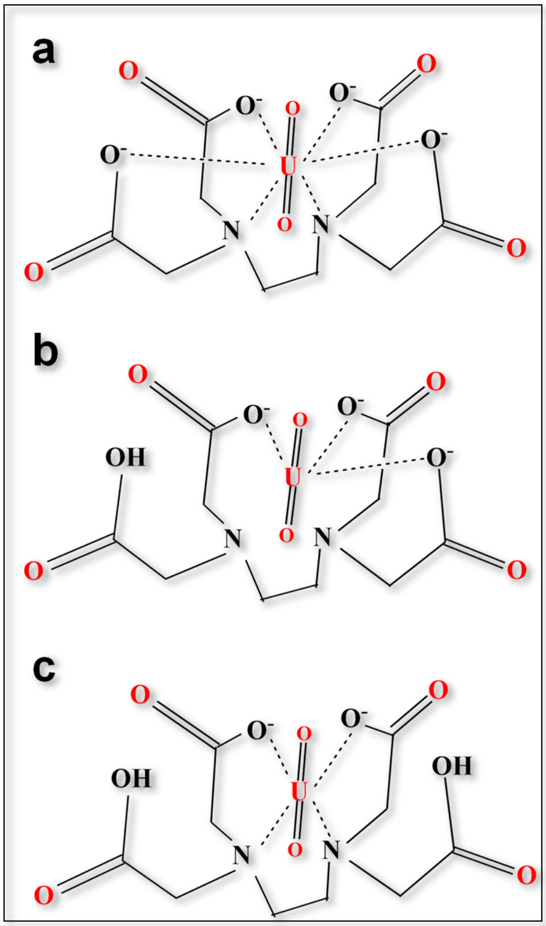

Figure S28. (a–c) Various possible coordination forms of UO_2^{2+} and EDTA.

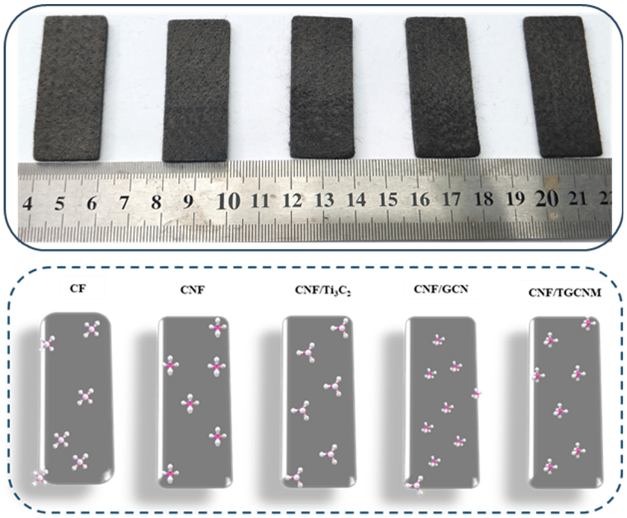

Figure S29. Digital photo of the as-prepared cathodes

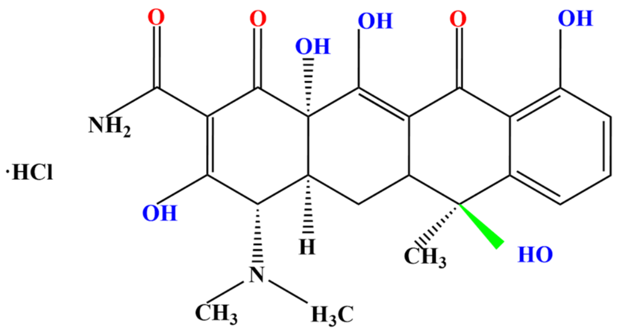

Figure S30. Molecular structure of Bisphenol A (BPA).

Table S1. Surface area, average pore size and pore volume of different materials.

Materials *(Photo-cathode/Anode)*	Surface area ($m^2 \cdot g^{-1}$)	Average pore size (nm)	Pore volume ($cm^3 \cdot g^{-1}$)
**TGCNM**	531.45	4.47	0.55
**GCN**	52.52	17.51	0.23
**P-MOF**	34.19	24.56	0.25
**Ti_3C_2 MXene**	41.53	10.30	0.11

Table S2. Power generation performance of different cathodes in UO_2^{2+} and BPA removal systems. Conditions: C_0 (UO_2^{2+}) = 10 mg·L^{-1}, C_0 (BPA) = 10 mg·L^{-1}, pH = 5, T = 298K, AM 1.5 illumination.

Cathode	J_{SC} (mA·cm^{-2})	V_{OC} (V)	P_{max} (mW·cm^{-2})	FF (%)
**GCN**	1.09	2.03	1.08	48.82
**PET-MOF**	1.23	2.13	1.27	46.20
**Ti_3C_2@P-MOF**	1.42	2.25	1.53	47.88
**Ti_3C_2 MXene**	1.32	1.96	1.15	44.57
**Ti_3C_2@GCN/P-MOF**	1.43	2.38	1.82	53.52

Table S3. Electricity generation performance of ALCE-GCN and ALCE-TGCNM in UO_2^{2+} and BPA removal system. Conditions: C_0 (UO_2^{2+}) = 10 mg·L^{-1}, C_0 (BPA) = 10 mg·L^{-1}, pH = 5, T = 298K, AM1.5 illumination.

Cathode	J_{SC} (mA·cm^{-2})	V_{OC} (V)	P_{max} (mW·cm^{-2})	*FF* (%)
**GCN**	1.09	2.03	1.08	48.82
**TGCNM**	1.43	2.38	1.82	53.52

Table S4. Effects of the electrolyte concentration on the electricity production of ALCE-TGCNM. Conditions: C_0 (UO_2^{2+}) = 10 mg·L^{-1}, C_0 (BPA) = 10 mg·L^{-1}, pH = 5, T = 298 K, AM 1.5 illumination.

C_0 (Na_2SO_4, mol·L^{-1})	J_{SC} (mA·cm^{-2})	V_{OC} (V)	P_{max} (mW·cm^{-2})	*FF* (%)
0	0.09	2.33	0.05	23.84
0.02	1.10	1.96	0.95	44.18
0.05	1.12	2.07	1.12	47.25
0.1	1.43	2.38	1.82	53.52
0.5	1.72	1.99	1.51	44.15

Table S5. Effects of the solution pH on the electricity production of ALCE-TGCNM Conditions: C_0 (UO_2^{2+}) = 10 mg·L^{-1}, C_0 (BPA) = 10 mg·L^{-1}, pH = 5, T = 298 K, AM 1.5 illumination.

pH	J_{SC} (mA·cm^{-2})	V_{OC} (V)	P_{max} (mW·cm^{-2})	*FF* (%)
2.0	1.59	2.46	1.49	38.09
3.1	1.78	2.50	1.68	37.75
4.0	1.75	2.48	1.64	37.78
5.0	1.43	2.38	1.82	53.52
5.9	1.64	2.33	1.52	39.79
7.1	1.62	2.48	1.43	35.66
8	1.75	2.47	1.63	37.73

Table S6. Effects of the initial BPA concentration on the electricity production of ALCE-TGCNM. Conditions: C_0 (UO_2^{2+}) = 10 mg·L^{-1}, pH = 5, T = 298 K, AM 1.5 illumination.

**BPA (mg·L^{-1})**	J_{SC} **(mA·cm^{-2})**	V_{OC} **(V)**	P_{max} **(mW·cm^{-2})**	*FF* **(%)**
5	1.25	2.41	1.50	49.83
10	1.43	2.38	1.82	53.52
20	1.38	2.43	1.73	51.59
40	1.05	2.37	1.35	54.26

Table S7. Effects of the initial UO_2^{2+} concentration on the electricity production of ALCE-TGCNM. Conditions: C_0 (BPA) = 10 mg·L^{-1}, pH = 5, T = 298 K, AM 1.5 illumination.

**UO_2^{2+} (mg·L^{-1})**	**J_{SC} (mA·cm^{-2})**	**V_{OC} (V)**	**P_{max} (mW·cm^{-2})**	**FF (%)**
5	1.11	2.36	1.33	50.78
10	1.43	2.38	1.82	53.52
20	1.15	2.35	1.42	52.54
40	1.51	2.33	1.75	49.85

Table S8. Effects of the anions on the electricity production of ALCE-TGCNM. Conditions: $C_0 (UO_2^{2+})$ = 10 $mg \cdot L^{-1}$, C_0 (BPA) = 10 $mg \cdot L^{-1}$, pH = 5, T = 298 K, AM 1.5 illumination.

**Anions**	J_{SC} **(mA·cm⁻²)**	V_{OC} **(V)**	P_{max} **(mW·cm⁻²)**	*FF* **(%)**
**Control**	1.43	2.38	1.82	53.52
**Cl^-**	1.85	2.31	1.60	38.09
**Br^-**	1.96	2.33	1.70	37.23
**NO_3^-**	1.48	2.40	1.45	40.82
**HCO_3^-**	1.75	2.33	1.54	37.77
**$H_2PO_4^-$**	1.92	2.30	1.63	36.96

Table S9. Effects of the cations on the electricity production of ALCE-TGCNM. Conditions: $C_0\,(UO_2^{2+})$ = 10 mg·L^{-1}, C_0 (BPA) = 10 mg·L^{-1}, pH = 5, T = 298 K, AM 1.5 illumination.

Cations	J_{SC} (mA·cm^{-2})	V_{OC} (V)	P_{max} (mW·cm^{-2})	*FF* (%)
**Control**	1.43	2.38	1.82	53.52
**Mg^{2+}**	1.60	2.44	1.62	41.53
**Zn^{2+}**	1.60	2.43	1.58	40.63
**Cd^{2+}**	1.66	2.36	1.52	38.87
**Ni^{2+}**	1.34	2.39	1.34	41.87
**Ca^{2+}**	1.60	2.39	1.51	39.48
**Fe^{3+}**	1.35	2.47	1.30	39.03
**Cu^{2+}**	1.53	2.40	1.51	41.14

Table S10. Simulate the concentration of ions in uranium-contaminated seawater.

**Ionic salts**	**Concentration**
**UO_2^{2+} (mg L^{-1})**	5
**NaCl (g L^{-1})**	26.5
**$MgCl_2$ (g L^{-1})**	24
**KCl (g L^{-1})**	0.73
**$MgSO_4$ (g L^{-1})**	3.3
**$NaHCO_3$ (g L^{-1})**	0.2
**$CaCl_2$ (g L^{-1})**	1.1
**NaBr (g L^{-1})**	0.28

Table S11. The electricity production performance of ALCE-TGCNM in treating simulated seawater.

Cathode	J_{SC} (mA·cm^{-2})	V_{OC} (V)	P_{max} (mW cm^{-2})	FF (%)
**Simulated seawater**	1.53	2.46	1.55	41.22

Table S12. The electricity production performance of ALCE-TGCNM in treating different organic pollutants - uranium contaminated wastewater. Conditions: $C_0\,(UO_2^{2+})$ = 10 mg·L^{-1}, C_0 (organic) = 10 mg·L^{-1}, pH = 5, T = 298K, AM 1.5 illumination.

Pollutants	J_{SC} (mA·cm^{-2})	V_{OC} (V)	P_{max} (mW·cm^{-2})	FF (%)
**CIP**	1.65	2.30	1.40	36.93
**SMX**	1.42	2.25	1.36	39.65
**ATZ**	1.59	2.41	1.52	40.12
**IBP**	1.49	2.31	1.36	38.23
**PNP**	1.65	2.28	1.44	37.40

Table S13. Effects of the initial EDTA concentration on the electricity production of ALCE-TGCNM. C_0 (UO_2^{2+}) = 10 mg·L^{-1}, under AM 1.5 illumination.

**EDTA (mg·L^{-1})**	J_{SC} **(mA·cm^{-2})**	V_{OC} **(V)**	P_{max} **(mW·cm^{-2})**	***FF*** **(%)**
5	1.42	2.09	1.37	46.28
20	1.41	2.07	1.12	38.48
50	1.45	2.01	1.16	39.86

Table S14. Comparison of removal efficiency with previously reported materials in terms of uranium extraction and BPA removal performance.

Methods	Anode	Cathode	Time (min)	C_0 (UO_2^{2+} mg·L^{-1})	C_0 (Organic pollutants)	Removal efficiency of organic pollutants (%)	Removal efficiency of UO_2^{2+} (%)	Ref.
Adsorption method (adsorbent)	MnO/MXene		4	10	--	--	99.70	[35]
	ZVI (P-ZVIbm)		120	10	--	--	90.00	[36]
	PPN-3		40	20	--	--	95.00	[37]
	$CNFe_2O_4$		60	50	--	--	98.00	[38]
Photocatalysis (photocatalyst)	TiO_2-PAN		120	100	--	--	91.58	[39]
	pTTT-Ben		600	200	--	--	80.00	[40]
	Zr-Fc-MOF3		140	4	--	--	95.70	[41]
Capacitive deionization (CDI)	PDA@MoS_2		330	50	ACT	92.18	--	[42]
	PPy@$MoSe_2$@MXene		150	300	PFS	60.98	--	[43]
Photo-electrocatalyti-c removal	Ni@C-700		120	10	--	--	93.90	[44]
Photo-Fenton process	Fe-Ce-Co		180	5	SMZ	--	90.62	[45]
	Co/C		90	10	--	86.32	--	[46]
**ALCE**	**CNF**	**TGCNM**	**120**	**10**	**10**	**97.70**	**96.70**	**This work**

Table S15. Pseudo-first-order and pseudo-second-order kinetic fitting parameters for U(VI) removal and BPA degradation.

Target	Kinetic model	k	R^2
U(VI) removal	Pseudo-first-order	0.061 min^{-1}	0.992
U(VI) removal	Pseudo-second-order	0.044 min^{-1}	0.941
BPA degradation	Pseudo-first-order	0.052 min^{-1}	0.988
BPA degradation	Pseudo-second-order	0.037 min^{-1}	0.919

Table S16. Structural and performance stability of the TGCNM cathode following five consecutive regeneration cycles using 0.1 M $NaHCO_3$ elution.

Parameter	Value
Number of regeneration cycles tested	15
U(VI) removal retention	93.9%
BPA removal retention	94.3%
Cumulative cathode mass loss (ICP-based)	< 4.8%
New XRD diffraction peaks after cycling	None observed
Elution agent	0.1 M $NaHCO_3$

Text S5. Materials

The precursor material, titanium aluminum carbide (Ti_3AlC_2, 200 mesh), was procured from Jilin Yiyi Technology Co., Ltd. (Jilin, China). Chemical reagents including Hydrogen fluoride (HF), hydrochloric acid (37 wt%), Polyvinylpyrrolidone (PVP, Mw 55,000), 1,3,5-benzenetricarboxylic acid ($C_9H_6O_6$, 99%), bisphenol A (BPA), and thioacetamide (TAA) were sourced from Aladdin Biochemical Technology Co., Ltd. Commercial carbon felt (CF) with the following specifications was obtained from Tianjin Carbon Plant (China): thickness 3 mm, porosity 90%, ash content ≤ 200 ppm, density 0.13-0.16 $g \cdot cm^{-3}$, hygroscopicity 1.0%, and thermal conductivity ranging from 0.27 $W \cdot m^{-1} \cdot K^{-1}$ at 20°C to 0.46 $W \cdot m^{-1} \cdot K^{-1}$ at 1000°C. Uranyl nitrate hexahydrate ($UO_2(NO_3)_2 \cdot 6H_2O$) was supplied by Macklin Biochemical Co., Ltd. (Shanghai, China).

Text S6. Preparation of Photocathode

(a) Synthesis of g-C_3N_5

A 10 mL pressure glass tube was charged with 1,3,5-trialdehyde phenyltriazine (25 mg, 0.065 mmol), 0.5 mL mesitylene, 0.5 mL 1,4-dioxane, and 100 μL aqueous 6 M acetic acid. To the suspension, hydrazine hydrate (5 μL, 50–60% solution) was then added as per previous studies[31]. The tube was then sealed and heated in a blast oven at 120 °C for 3 d at autogenous pressure. Thereafter, the tube was opened and the suspension was filtered and washed with chloroform (2 × 5 mL), acetone (2 × 5 mL), and tetrahydrofuran (2 × 5 mL). The solid was dried at room temperature under a vacuum to afford TGCNM as a light-yellow powder (Yield: 76.2%, 18.9 mg).

(b) Synthesis of Ti_3C_{2x}

In the synthesis process through direct etching procedure, 5g of Ti_3AlC_2 was added gradually to a 100 mL of 50 wt% hydrofluoric acid (HF) while stirring using Teflon magnetic bar. Slow addition of MXene powder is necessary to minimize excessive bubbling formed due to the exothermic reaction. The etching process was then allowed to proceed at room temperature (~23ºC) for 5 h under stirring at 300 rpm. The sample then be separated using centrifugation (5 min per cycle at 3500 rpm) and washed with deionized water until the pH value is almost neutral. The pH value was determined by using a pH paper indicator. The sediments were re-washed with 1000 mL of deionized water via vacuum-assisted filtration using filter membrane with 0.22 μm pore size filters. The sample let to dry in vacuum oven for 24 h at 80˚C.

Synthesis of P-MOF

(a) *Synthesis of Waste PET-derived BDC*

The process schematic for the synthesis of terephthalic acid derived from PET according to reference is illustrated in **Scheme S1**. Briefly, the clear mineral water bottles' labels and caps were removed, and the bottles were cut into small pieces (~3–5 mm). The small flakes were washed with water and soap before being dried in an oven at 60 °C overnight. Then, the PET hydrolysis experiment was carried out in a stainless-steel autoclave lined with Teflon. PET flakes (2.5 g) were placed in the autoclave along with 5 mL of ethylene glycol (EG) and 50 mL of H_2O, and the reactor was heated to 210 °C and kept there for 8 h. After the reaction, the product was centrifuged, washed twice with ethanol, and dried at 80 °C for 12 h. The residual PET was dried at 70 °C until constant weight was achieved. PET conversion was calculated as 87% using the formula.

$$Conversion\ of\ PET(\%) = \frac{(Initial\ weight\ of\ PET\ -\ Weight\ of\ residual\ PET)}{(Initial\ weight\ of\ PET)} \times 100$$

(b) Synthesis of Ti-MOF derived PET-BDC

Terephthalic acid (TPA) employed in this study was recycled from polyethylene terephthalate plastic bottles using solvothermal method reported previously. The recycling process is summarized in **Scheme S1**. The purity of recovered TPA, as reported previously, was 98% and was used for the preparation of MOF without further purification. The procedure for the synthesis of MOF by a hydrothermal method, as reported in previous studies. Briefly, a mixture of a 1:1 stoichiometric ratio of 1.332 g (1 mmol) of $TiCl_2.2H_2O$ and 0.182 g (1 mmol) of PET which is dissolved in the ethanol under sonication with NaOH/BDC=2 for deprotonation. 8 g (2 mmol) of NaOH and 0.182 g (1 mmol) of PET-BDC sonicated in EtOH to form suspension on linker solution in a drop-wise manner was then heated at 170 °C/24 h and the resultant solution was subjected to EtOH/MeOH washing followed by centrifugation. The obtained PZM@BC solid residue was then cleansed, initially by washing with EtOH and centrifuging to eradicate surplus NaOH until the pH reached around neutral, and then through vacuum filtration and drying in a hot air oven for 100°C at 3 h.

Text S7. Preparation of Photoanodes (CNF)

The Ti_3C_2 seed layer was deposited on an FTO-coated glass substrate using the following method. First, the substrate (2.2 mm thick, functional area of 2×4 cm^2, and a resistance of 13.3 Ω/cm) was cleaned by ultrasonication in acetone and deionized water for 30 minutes. Next, a solution was made by mixing 0.2 mL of Ti_3C_2 with 20 mL of ethanol in a beaker. This mixture was stirred at 600 rpm for 30 minutes at room temperature (25°C). The solution was then spin-coated onto the FTO substrate at 3000 rpm for 30 seconds, dried at 60°C for 10 hours, and annealed at 450°C for 2 hours.

Ti_3C_2 nanorod arrays were synthesized through a solvothermal growth mechanism. Initially, a 1:1 (v/v) hydrochloric acid solution was formulated by blending 30 mL deionized water with 30 mL concentrated HCl (37 wt%), followed by mechanical agitation (500 rpm, 10 min). MXene was incrementally introduced into the acidic medium under enhanced stirring (600 rpm, 10 min). The homogeneous precursor was transferred to a 100 mL Teflon-lined autoclave containing an FTO substrate (seed layer orientation: downward-facing), which underwent thermal treatment at 170 ± 5°C for 6 h (heating rate: 5°C/min). Post-synthesis processing included sequential rinsing cycles (DI water, 3×), vacuum desiccation (80°C, 5 h), and phase crystallization via thermal annealing (500°C, 2 h, air atmosphere) to achieve vertically aligned rutile-phase structures.

Text S8. Structure and Properties of Photoanode

Scanning electron microscopy (SEM) characterization of the Carbon/chitosan Nano film (CNF) arrays disclosed a well-organized architecture with perpendicularly oriented nano cube assemblies on fluorine-doped tin oxide (FTO) substrates (**Figure S9**). X-ray diffraction analysis comparing the nano cube film (upper) and bare FTO (lower), as illustrated in **Figure S10**, identified dominant crystalline reflections assigned to the (101) and (002) lattice planes of rutile-phase of carbon/chitin film [32]. The exclusion of extraneous peaks (e.g., (110), (111), (211)) confirmed single-crystalline growth with preferential crystallographic alignment, consistent with SEM morphological observations [23].
